# Inertness of Superoxide Dismutase Mimics Mn(II) Complexes Based on an Open-Chain Ligand, Bioactivity, and Detection in Intestinal Epithelial Cells

**DOI:** 10.1155/2022/3858122

**Published:** 2022-04-01

**Authors:** Gabrielle Schanne, Martha Zoumpoulaki, Géraldine Gazzah, Amandine Vincent, Hugues Preud'homme, Ryszard Lobinski, Sylvie Demignot, Philippe Seksik, Nicolas Delsuc, Clotilde Policar

**Affiliations:** ^1^Laboratoire des Biomolécules, LBM, Département de chimie, Ecole Normale Supérieure, PSL University, Sorbonne Université, CNRS, 75005 Paris, France; ^2^Centre de Recherche Saint Antoine, INSERM, UMRS 938, Metabolism-Inflammation Department, 184 Rue du Faubourg Saint-Antoine, 75012 Paris, France; ^3^IPREM-UMR5254, E2S UPPA, CNRS, Technopôle Helioparc, 2 Avenue P. Angot 64053 Pau Cedex 9, France; ^4^EPHE, PSL University, 75014 Paris, France; ^5^Gastroenterology Department, Saint-Antoine Hospital, Sorbonne Université, APHP, Paris, France

## Abstract

Oxidative stress is known to play a major role in the pathogenesis of inflammatory bowel diseases (IBDs), and, in particular, superoxide dismutase (SODs) defenses were shown to be weakened in patients suffering from IBDs. SOD mimics, also called SOD mimetics, as low-molecular-weight complexes reproducing the activity of SOD, constitute promising antioxidant catalytic metallodrugs in the context of IBDs. A Mn(II) complex SOD mimic (Mn1) based on an open-chain diaminoethane ligand exerting antioxidant and anti-inflammatory effects on an intestinal epithelial cellular model was shown to experience metal exchanges between the manganese center and metal ions present in the biological environment (such as Zn(II)) to some degrees. As the resulting complexes (mainly Zn(II)) were shown to be inactive, improving the kinetic inertness of Mn(II) complexes based on open-chain ligands is key to improve their bioactivity in a cellular context. We report here the study of three new Mn(II) complexes resulting from Mn1 functionalization with a cyclohexyl and/or a propyl group meant to limit, respectively, (a) metal exchanges and (b) deprotonation of an amine from the 1,2-diaminoethane central scaffold. The new manganese-based SOD mimics display a higher intrinsic SOD activity and also improved kinetic inertness in metal ion exchange processes (with Zn(II), Cu(II), Ni(II), and Co(II)). They were shown to provide anti-inflammatory and antioxidant effects in cells at lower doses than Mn1 (down to 10 *μ*M). This improvement was due to their higher inertness against metal-assisted dissociation and not to different cellular overall accumulations. Based on its higher inertness, the SOD mimic containing both the propyl and the cyclohexyl moieties was suitable for intracellular detection and quantification by mass spectrometry, quantification, that was achieved by using a ^13^C-labeled Co-based analog of the SOD mimics as an external heavy standard.

## 1. Introduction

Superoxide dismutases (SODs) are metalloenzymes that catalyze very efficiently the superoxide dismutation at a rate close to the diffusion limit [1]. Superoxide is one of the reactive oxygen species (ROS) and is mainly produced at the mitochondria as a byproduct of the respiratory metabolism in living aerobic systems. SODs are responsible for maintaining superoxide at tightly controlled levels and contribute to prevent oxidative stress situations known to be implicated in various diseases [2]. In their active site, all of the SODs contain a metal cation (Cu(Zn), Cu, Fe, Mn, or Ni) that cycles between two redox states to successively reduce superoxide to H_2_O_2_ and to oxidize it into O_2_. Three different kinds of human SODs have been described: two copper/zinc SODs, SOD1 found in the cytosol and in the mitochondrial intermembrane space [3] and SOD3 found in the extracellular environment [4] (within the extracellular matrix and at cell surface), and the manganese SOD (SOD2) localized in the mitochondria matrix [5]. Inflammatory bowel diseases (IBDs) including Crohn's disease and ulcerative colitis are accompanied by an overexpression of the intestinal MnSOD in an enzymatically inactive form and by an underexpression of the intestinal cytoplasmic Cu/Zn SOD [6]. These deficiencies in the SOD antioxidant system may cause or, at least, exacerbate the oxidative stress observed in IBDs which is known to be closely related to chronic inflammation [7–9]. A SOD-based antioxidant treatment thus appears as a promising therapy for IBDs. MnSODs were shown to efficiently reduce lipid peroxidation and neutrophil recruitments and to attenuate the inflammation in both DSS-induced and TNBS-induced colitis murine models [10]. However, the use of purified enzymes as therapeutics is still limited by their short half-life, the triggered immunogenicity, and their low cell penetration [11]. To overcome these shortcomings, the use of synthetic low-molecular-weight SOD mimics, also called SOD mimetics, was examined as therapeutic candidates for IBD management [11, 12].

A large variety of SOD mimics have been reported including iron, copper, zinc, and manganese complexes [11–20]. Manganese complexes are favored in comparison with Cu, Fe, and Ni complexes since manganese, if released, is better tolerated by cells and does not catalyze the Fenton or Haber-Weiss reactions that lead to the formation of the extremely reactive and toxic HO^∙^ radical [21]. Manganese SOD mimics described in the literature include complexes with ligands such as porphyrins [14, 22–28], salens [29, 30], cyclic polyamines [11, 31–37] phthalocyanines [38], or peptidyl ligands [39–41]. We have been developing a 1,2-ethanediamine-centered ligand, called EnPI2 [42], inspired by the MnSOD-active site, with a coordination sphere consisting of three imidazoles and one phenolate, easily amenable to synthetic modulation [43–46]. Mn(II)-EnPI2, labeled Mn1, is a positively charged complex with a redox potential close to the optimal value for superoxide dismutation [42, 44]. Mn1 shows a clear anti-superoxide activity out of any cellular context, as well as antioxidant and anti-inflammatory effects on intestinal epithelial cells and macrophages [44, 47, 48]. Mn1 also ameliorates DNBS-induced colitis in a murine model according to the weight variation and macroscopic scores [44].

The structure of EnPI2 can be optimized to improve the stability and, especially, the inertness of the derived Mn(II) complex since Mn decoordination in cells was suggested in a previous study [46]. Indeed, given the symmetrical d^5^ electronic configuration of the Mn^2+^ ion, the corresponding complexes lack ligand field stabilization energy and are much less stable than other divalent transition metal analogues (Ni^2+^, Cu^2+^, Co^2+^, Zn^2+^, etc.). Consequently, many manganese complexes, particularly with open-chain ligands such as EnPI2, may suffer from dissociation [49]. In a recent paper, we have investigated speciation of a complex derived from Mn1 based on a ligand tagged with a Re probe: the Mn and Re X-fluorescence maps, recorded above the edges of both Re and Mn, were overlapping, but only partly: a few areas showed only Re, revealing indirectly some dissociations have occurred in cells [44]. The dissociation may occur via different pathways, such as spontaneous decoordination and acid-catalyzed or metal-assisted decomplexation involving Mn^2+^ exchange with a competitive metal ion. However, in biological systems, the endogenous ligands competing for Mn(II) binding also have low association constants, rendering the criteria on the association constant paradoxically less drastic for Mn(II) than for other metal cations [15, 44].

To date, the cellular accumulation of SOD mimics was usually examined by measuring the increase in the overall intracellular content in manganese using ICP-MS [46, 50–52], EPR [44–46], Raman [53], X-ray absorption spectroscopy [36], and X-ray fluorescence imaging [13, 36, 44–46] or by detecting the cellular uptake using UV quantification of Mn porphyrin via the Soret band area [16, 54] or of fluorescently labeled SOD mimics using laser confocal microscopy [36, 40, 46, 55]. At best, these techniques enable determining the intracellular distribution of the ligand, complexes, and/or Mn. In the latter case, the differential distribution of Mn from that of endogenous Mn provides insight on the Mn complex distribution [13, 36, 44–46]. However, for the bioinspired series developed in our group, even if the MS-MS spectrum of Mn1 was observed in cell lysates [42], no direct information on the speciation and metal exchanges or quantification in cell lysates of the intact complexes has been reported [15, 41, 43–47].

As mentioned above, a higher inertness of the manganese complexes would be an important asset for improving the bioactivity of the SOD mimics and make them more suitable for *in vivo* application. Open-chain ligands derived from EDTA containing a cyclohexyl group bearing the diamino moiety in order to rigidify the structure of the ligand, with appended carboxylate (CDTA) or pyridine (PyC3A) (see Figure S1), have been developed by Kálmán and Tircsó and Gale et al. (Figure S1) [49, 56]. The [Mn(II)-(CDTA)]^2+^ complex revealed a highly improved resistance to dissociation *via* metal exchanges [49]. This behavior was explained by the formation of a preorganized and more rigid coordination cavity that encapsulates the manganese ion. Other open-chain ligands for Mn coordination were described, but they did not reach as good kinetic inertness as Mn-CDTA [57]. Mn-CDTA complex is hence considered as the gold standard for rigid and inert Mn^2+^ complex involving an open-chain ligand. Only very recently, Ndiaye et al. succeeded in synthetizing a new Mn^2+^ bispidine chelate with unprecedented kinetic inertness [58]. Inspired by these strategies, we have replaced the 1,2-diamino group of EnPI2 by a (±)-trans-1,2-cyclohexyl diamine to rigidify the structure and hopefully increase the resulting SOD mimic inertness. Stability, inertness, and more generally bioactivity of the corresponding complexes have been investigated. N-Propylated analogs of Mn1 and of its cyclohexyl analogue were also investigated and evaluated in a cellular model consisting of intestinal epithelial cells (HT29-MD2) activated with bacterial lipopolysaccharide (LPS) [59]. We have compared the ability of these Mn1-derived SOD mimics to limit oxidative stress and inflammation when incubated at different doses ranging from 0.1 *μ*m to 100 *μ*M. We propose to use high-definition mass spectrometry to detect unambiguously one of these complexes in cell lysates and to quantify their amount by using a ^13^C-labeled analog as an external standard.

## 2. Material and Methods

### 2.1. Synthesis of the Ligands

See supplementary information.

### 2.2. Titration of the Ligands by UV Experiments

The concentration of the ligands in solution was determined by a UV-vis titration experiment. Small quantities of MnCl_2_ were successively added to a solution of ligand estimated at ca. 200 *μ*M (in HEPES 50 mM, pH 7.5), and the absorbance of the Mn(II) complexes was monitored at 288 nm (MLCT O_phenolate_ → Mn) [44]. The concentration of ligand was calculated from the amount of added MnCl_2_ at the equivalence.

### 2.3. Preparation of the Complexes for Experiments Other than Cellular Assays

**Figure 1:**
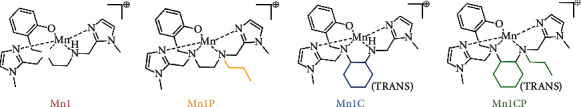
Structures of the manganese (II) complexes mentioned in the text.

The manganese complexes Mn1, Mn1P, Mn1C, and Mn1CP (Figure 1) were prepared by the addition of 1 equivalent of MnCl_2_ to a solution of the ligand (pretitrated as explained above) in HEPES buffer (50 mM, pH 7.5).

### 2.4. Association Constants

The association constants of the manganese complexes were determined by UV-vis titration experiments. The absorbance at 288 nm was monitored while adding successively 0.1 equivalent of manganese to a solution of ligand at 45 *μ*M in HEPES (50 mM, pH 7.5). The initial concentration in ligand was chosen lower than 100∗*K*_d_ known for Mn1 (ca. 7.57·10^−7^ M [44]) in order to observe an equilibrium between uncoordinated ligand L, Mn^2+^, and the complex in solution at the 1 : 1 Mn : L ratio and obtain not only the stoichiometry but also the association constant (C_ligand_ should be smaller than 100∗*K*_d_; see caption of Figure S2) [60]. The association constant was then obtained by fitting the theoretical absorbance curve to the experimental one using the MATLAB curve fitting tool (Figure S2). The calculations are detailed in the supplementary information. The association constants of the Zn(II) and Co(II) complexes are too high to be determined using the same protocol, as they would require a too weak concentration to meet the criterion of an equilibrium at the 1 : 1 ratio, too weak to follow the complexation using UV-vis. Therefore, we performed competition experiments between the Mn(II) and Zn(II) or Co(II) complexes. For Zn(II) complexes, solutions of manganese complexes prepared at 10 *μ*M with 200 equivalents of MnCl_2_ in HEPES (50 mM, pH 7.5) were titrated with ZnCl_2_ solution (Figure S19). For Co1CP, a solution of Mn1CP prepared at 40 *μ*M with 50 equivalents of Mn (in HEPES 50 mM, pH 7.5) was titrated with CoCl_2_ solution (Figure S28). The association constants of the zinc or cobalt complexes were then similarly obtained by fitting the theoretical absorbance curve to the experimental one using the MATLAB curve fitting tool (Figure S19 and Figure S28). The calculations are detailed in the supplementary information.

### 2.5. Kinetic Study of the Metal Exchanges by UV Spectroscopy

A solution of each of the SOD mimics was prepared at 0.1 mM in Tris buffer (50 mM, pH 7.5) in a semi-microcuvette (1.5 mL). One equivalent of competitive metal salts (ZnCl_2_, CuSO_4_, NiCl_2_·6H_2_O, or CoCl_2_) was then added in the microcuvette, and, after a quick manual stirring, the absorbance at a specific wavelength was measured for about 10 min. The wavelengths were chosen to have a high difference in absorbance between the manganese complex and the exchanged metal complex (Figures S3, S4, S5, and S6). For Cu^2+^, Zn^2+^, and Co^2+^, the experiments were performed at 5°C as the metal exchanges were too fast at 25°C preventing any kinetic study. For Ni^2+^ exchange study, the temperature was maintained at 25°C.

### 2.6. Kinetic Study of the Metal Exchanges Using a Stopped-Flow Technique

Solutions of SOD mimics and competitive metal ions were prepared, respectively, at 300 *μ*M and at 4 mM in Tris buffer (50 mM buffer, pH 7.5). The solutions of SOD mimic and competitive metal ion were added in two different syringes (*V* = 10 mL) of the stopped-flow apparatus, and a similar mixing sequence was used for each analysis. A total volume of 226 *μ*L was injected with a flow rate of 13 mL/s: 88 *μ*L of the SOD mimic solution and 138 *μ*L of the competitive metal solution. After mixing in a high-density mixer, the SOD mimic is thus at 120 *μ*M and the competitive metal ions at 2400 *μ*M (20-fold excess). The metal exchanges were monitored at ambient temperature by UV-vis spectroscopy between 200 nm and 400 nm. The pseudo-first-order rate was then obtained by fitting the theoretical time-absorbance curve to the experimental one using the Biokine32 software.

### 2.7. Electrochemistry

Cyclic voltammetry experiments were performed with manganese(II) complex solutions at 100 *μ*M in HEPES buffer (50 mM, pH 7.5, ionic strength = 12.5 mM). Experiments were carried out under an argon stream at room temperature. The auxiliary electrode was a Pt wire, and the working electrode was a glassy carbon or a platinum disk. The reference electrode was a SCE saturated with KCl.

### 2.8. Intrinsic SOD Activity

The catalytic rate (*k*_cat_) for superoxide dismutation with the SOD mimics was determined in HEPES buffer (50 mM, pH 7.4) using an indirect assay developed by McCord and Fridovich [15, 42, 61–63]. This assay is based on a competition between the SOD mimic and a redox marker, in our case XTT (2,3-bis-(2-methoxy-4-nitro-5-sulfophenyl)-2H-tetrazolium-5-carboxanilide), to react with superoxide that is continuously produced via an enzymatic xanthine (200 *μ*M)/xanthine oxidase system. In this assay, we used [XTT] = 100 *μ*M. The formation of formazan resulting from XTT oxidation by superoxide was monitored by UV spectroscopy. The *k*_cat_ of the SOD mimics were obtained from the IC_50_, concentration of SOD mimics required to reduce by 50% the formation of formazan and from the known value of *k*_cat_ of XTT [15, 64].

### 2.9. Cell Culture

HT29-MD2 intestinal epithelial cells were used for all experiments. HT29 human colon adenocarcinomas were obtained from the European Collection of Cell Cultures (Wiltshire, UK) and stably transfected to overexpress the protein MD2, a coreceptor of TLR4 necessary to confer sensitivity to LPS [59]. Cells were cultured in DMEM supplemented with 10% heat-inactivated FBS and blasticidin (10 *μ*g/mL) at 37°C in a 5% CO_2_/air atmosphere.

### 2.10. Cell Activation with LPS and Incubation with the SOD Mimics

Cells were seeded in 12-well plates (for cells assays) or in T75 flasks (for manganese and complex quantification) at 50,000 cells/cm^2^ and cultured until 90% confluence. Cells were coincubated for 6 hours with LPS at 0.1 *μ*g/mL and with the complexes at concentrations varying between 0.1 *μ*M and 100 *μ*M with a ratio ligand : Mn 1 : 1.4. An incubation time of 6 h was chosen based on previously published results on Mn1 intracellular quantification by EPR and for the detection of the inflammatory markers, IL8 and COX2 [44]. The titration of manganese content over time showed that Mn1 accumulation was stabilized after 3-4 hours. We can thus consider that the maximal accumulation is reached after 6 hours, time at which the inflammation markers were easily quantified after LPS challenge. Supernatants were collected and stored at −20°C until enzyme-linked immunosorbent assay (ELISA) and lactate dehydrogenase (LDH) assay were performed. For cell assays, the cells were washed with phosphate-buffered saline (PBS), lysed in PBS containing a 1% Triton X-100 and protease inhibitor cocktail, and sonicated. For total Mn and Mn complex quantification, the cells were washed with PBS, harvested in NH_4_HCO_3_ buffer (50 mM, pH 7.4) by scraping, and sonicated.

### 2.11. Cell Assays

Cell assays were conducted as previously described by Mathieu et al. and Vincent et al. [41, 44–46, 48]. Briefly, the cytotoxicity of the SOD mimics was assessed using the lactate dehydrogenase (LDH) assay. The protein concentrations were determined in cell lysates using bicinchoninic acid (BCA) protein assay reagents and bovine serum albumin (BSA) as the standard. The amount of interleukin-8 (IL-8) secreted in the cell supernatant was measured using a commercially available ELISA kit according to the instructions of the manufacturer (DuoSet). The IL8 levels were normalized by the protein content determined in the corresponding cell lysates. The expression of MnSOD in cell lysates was examined by Western blot. The abundance of MnSOD was normalized to the total amount of protein loaded using the stain-free imaging technology (Bio-Rad).

### 2.12. Quantification of the Mn Contents by ICP MS

The total Mn content was quantified in cell lysates by ICP MS. Cell lysates were acidified using 2% HNO_3_, to free Mn from all coordination sites, and filtered. A calibration curve was established using a commercial Mn standard. The total Mn amount was normalized by the mass of proteins in the cell lysate.

### 2.13. Detection and Quantification of Mn1CP in Cell Lysates by Mass Spectrometry

The cell lysates were ultracentrifuged at 100,000 rpm for 20 min and then diluted in 20% NH_4_CO_3_/80% ACN. For the quantification, an external standard was added at 0.4 *μ*M in the diluted cell lysates before analysis. The standard consisted of the cobalt(II) complex with EnPI2CP labeled with ^13^C on the phenol moiety obtained using CoCl_2_. The samples were then analyzed by TOF MS in direct infusion mode. Sample injection was performed with a 250 *μ*L syringe at 5 *μ*L/min flow rate. Acquisition was performed after 1 min, once the spray was stabilized, over 3-5 min. Between each measurement, the system was washed, successively with isopropanol, water, and isopropanol, to avoid contamination of the next sample and capillary blockage. To avoid metal contaminations, ultrapure solvents (acetonitrile, isopropanol, and water) and metal-free labware were used. A calibration curve was established by spiking control LPS-stimulated HT29-MD2 lysates with the Co1CP(^13^C) standard at 0.4 *μ*M and with Mn1CP at concentrations in the range 0.05 *μ*M to 0.8 *μ*M. The ratio between the signal intensity of Mn1CP and that of the standard was plotted as a function of Mn1CP concentration (Figure S29). For the calibration and quantification, the signal intensity of both Mn1CP and the standard was obtained by summing the peak intensities of the noticeable isotopes. This quantification based on the use of a heavy analog of Mn1CP as a standard was validated by the method of standard additions. For the latter, lysates of HT29-MD2 cells, beforehand incubated with Mn1CP at 100 *μ*M for 6 hours, were similarly ultracentrifuged at 100,000 rpm for 20 minutes, diluted in 20% NH_4_CO_3_/80% ACN and spiked with the standard Co1CP(^13^C) at 0.4 *μ*M. Before MS injection, Mn1CP was spiked at known concentration (0 *μ*M, 0.2 *μ*M, and 0.4 *μ*M) in the lysate. The ratio of the signal intensity of Mn1CP on that of the standard was plotted as a function of spiked Mn1CP concentration (Figure S31), and the equation of the linear regression gave us the estimated concentration in Mn1CP.

## 3. Results and Discussion

### 3.1. Design and Synthesis

Three new ligands derived from the parent EnPI2 were designed (Figure 1). For EnPI2C and EnPI2CP, a cyclohexyl group was added between the two amino groups in order to introduce rigidity into the ligand skeleton and consequently reduce the kinetic lability. As previously shown, the Mn complex [Mn(CDTA)]^2+^ or [Mn(PyC3A)]^2+^ studied, respectively, by Kálmán and Tircsó, Gale et al., and Laine et al., contains a similar diaminocyclohexane motif (Figure S1) and displayed remarkably slow dissociation rate, possibly due to a weaker ability to reach open conformation [49, 56, 57]. EnPI2-derived ligands were synthetized using a protocol similar to that used for EnPI2, previously reported by Cisnetti et al. [42] but with racemic (±)-(trans)-1,2-diaminocyclohexane instead of 1,2-diaminoethane as a reagent. Additionally, the secondary amines in EnPI2 and EnPI2C were propylated to obtain, respectively, EnPI2P and EnPI2CP. This allows to avoid the formation of a deprotonated form when studied in MS. The propylation was performed *via* a reductive amination using propionaldehyde.

For physicochemical characterization (not for cellular assays), the Mn^II^ complexes were prepared in situ by mixing the pretitrated ligand (see above) and MnCl_2_ in a 1 : 1 ratio in HEPES buffer (50 mM, pH 7.5), as previously described [44–46]. They were labeled Mn1, Mn1P, Mn1C, and Mn1CP depending on the associated ligand (respectively, EnPI2, EnPI2P, EnPI2C, and EnPI2CP) (Figure 1). Note that for the biological experiments, the SOD mimics were prepared with a ratio 1 : 1.4 ligand : Mn (see below).

### 3.2. Physicochemical Properties

Different criteria are required for efficient SOD mimics [11, 44]. They include a high catalytic activity of superoxide dismutation (or intrinsic activity), a good stability in biological environment, and the ability to penetrate inside cells to reach the site of action. The potentials of the four SOD mimics to fulfill these criteria were evaluated and compared.

#### 3.2.1. SOD Mimics Stability

*(1) Association Constants*. The association constants of the four manganese complexes in HEPES (50 mM, pH 7.5) were determined by UV-vis titration experiments, as shown in Figure S2, and the results are given in Table 1. The association constant of Mn1 was found to be (1.215 ± 0.176) × 106^6^ (M^−1^) which is consistent with the previously reported value determined by isothermal titration calorimetry [44] and UV-visible [65]. The inclusion of a cyclohexyl group on the diamino-central scaffold of both EnPI2 and EnPI2P structures was associated with a weak decrease of the ligands' association constant with Mn^2+^. The decrease can be explained by the stiffed structure of the ligands: the rigidity can induce enthalpically unfavorable constraints leading to a reduction in the association constant and also a slower complexation. The substitution on the secondary amine by a propyl group resulted also in a slight decrease of the association constant with Mn^2+^. A similar effect of N-alkylations on association constants was described by Martínez-Camarena et al. [66]: the presence of isopropyl substituents on the secondary amines of a tetra-azacyclophanes ligand was shown to produce a decrease in the stability of the corresponding copper complex. Riley et al. reported elsewhere that methyl substituents on cyclic polyamine ligands were not affecting clearly the association constant with Mn^2+^ [67], and previous functionalization with peptides showed the same weak effect of tertiarization of the secondary amine in EnPI2 [43]. The lower thermodynamic stability of manganese complexes compared to other divalent transition metal ions can be explained by the lack of ligand-field stabilization due to the symmetric d^5^ electron configuration system of the Mn^2+^ ion (Irving-Williams series) [44]. Coordinating molecules —Lewis bases— are abundant in biological media. However, considering the association constant for Mn^2+^, we have measured no ligand exchange with the competitive biomolecules that should take place in the cellular environment for any of the studied SOD mimics. Indeed, as mentioned above, the competitive endogenous ligands display low association constant with Mn^2+^, such as the archetypal bioligand: human serum albumin (*K*_1_ = 8.4 × 10^3^ M^−1^) [68]. This renders the criteria on the association paradoxically less drastic for Mn(II) than for other metal cations [15, 44]. The exchangeable pool of metal ions [69] contained in biological media could compete with the manganese(II) ion resulting in metal ion exchange and possibly loss of activity. However, it should be kept in mind that, although metal ions are overall abundant in cells (ca. 70 *μ*M for Cu, 0.001 to 10 *μ*M for Fe, and 180 *μ*M for Zn, for example) [70], metal ions are tightly controlled, either through coordination with proteic scaffolds (Zn, Cu) [69] or through precipitation (Fe in ferritin). Thus, the intracellular exchangeable pools of metal ions are much less concentrated (ca. 10^−10^ M for Cu (Cu(I)), 10^−11^ M for Zn) [69, 71]. Mn(II) has been classified as one of the most available metal cation (10^−5^ M for the bioavailable Mn(II)) [69], as opposite to Zn and Cu, classified as the most competitive [69].

All these features make the requirement for the stability of complexes less drastic for Mn complexes than for any other metal ion [15, 44, 69]. However, as metal ions exchange their ligand fast, we decided to explore the kinetic inertness of the SOD mimics that could be a crucial parameter for a better bioactivity.

**Figure 2:**
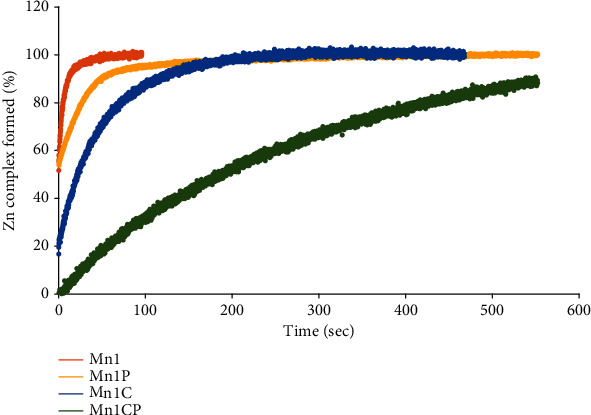
Kinetics study of the metal exchanges occurring between the manganese centers of the SOD mimics and with Zn^2+^. The percentage of complexes that underwent metal exchanges was monitored spectrophotometrically by following the absorbance at a wavelength, chosen to have a high difference in absorbance between the manganese complex and the Zn(II) complex. The spectra of the Mn(II) complexes and Zn(II) complexes are given in Figure S3 and were used to choose these monitoring wavelengths. The graphs presenting the kinetic of metal exchanges occurring between the Mn(II) centers of the SOD mimics and Cu^2+^, Ni^2+^, and Co^2+^ are given in Figure S7.

*(2) Kinetic Inertness*. The kinetics of the metal exchanges between manganese(II) and four biologically important divalent transition metal ions (Cu^2+^, Zn^2+^, Ni^2+^, and Co^2+^) added at one equivalent in a solution of each SOD mimic were monitored spectrophotometrically (Figure 2 and Figure S7). As predicted by the Irving-Williams series, metal exchanges take place in all cases until formation of 100% of the more stable competitive metal complex [72, 73]. To characterize the kinetic inertness of the Mn complexes, the half-lives (*t*_1/2_) of the transmetallation 1 : 1 reaction were determined for each experiment (Table 2). As shown in Table 2 and Figure 2, the exchanges are clearly slowed down for Mn1C and Mn1CP compared to Mn1 and Mn1P. The comparison of the *t*_1/2_ reveals that the inertness of Mn1CP differs by orders of magnitude in some cases depending on the nature of the competing metal ion. This qualitative approach confirms that EnPI2C and EnPI2CP constitute rigid open-chain ligands that efficiently reduce the kinetic lability of the manganese complexes [49]. In the case of Zn^2+^ or Cu^2+^ ions, the propylated complexes display higher *t*_1/2_ and thus increased kinetic inertness compared to the corresponding nonpropylated complexes. A similar trend was observed by Riley et al. who observed that the number of hydrocarbon substituents on cyclic polyamine ligands generally increases the kinetic inertness of the corresponding manganese complexes to dissociation [67]. However, this effect of the propylation is not seen for Ni^2+^ and Co^2+^ metal exchanges.

**Figure 3:**
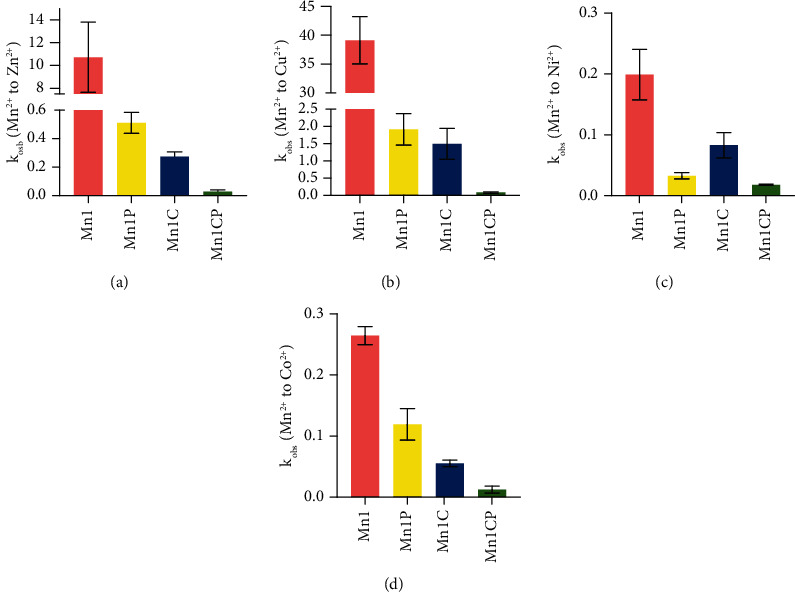
Pseudo-first-order rate constants *k*_obs_ (s^−1^) characterizing the metal exchanges occurring between the manganese centers of the SOD mimics with (a) Zn^2+^, (b) Cu^2+^, (c) Ni^2+^, and (d) Co^2+^. Kinetics of the exchanges were monitored in Tris (50 mM, pH 7.5) using a stopped-flow technique in the presence of 20-fold excess of competitive metal in order to ensure pseudo-first-order conditions. The exchanges were observed spectrophotometrically at room temperature and at specific wavelengths chosen to have a high difference in absorbance between the manganese complex and the competitive metal complex. The pseudo-first-order rates were then obtained by fitting the theoretical time-absorbance curve to the experimental one using the Biokine32 software. The numerical values of the *k*_obs_ are given in Table S1. Data represent the mean ± SEM for three independent experiments, and each independent experiment was performed in duplicates.

To ensure pseudo-first-order conditions and thus retrieve pseudo-first-order rate constants, the competitive metal ions have to be in large excess compared to the SOD mimic. However, in such conditions, the exchanges are too fast to be monitored by classical UV-vis techniques with manual mixing, particularly for Zn^2+^ and Cu^2+^. Stopped-flow analysis was then conducted to study the kinetics of metal exchanges when competitive metal ions were in 20-fold excess. The obtained pseudo-first-order rate constants (*k*_obs_) are given in Figure 3 (and Table S1). The new SOD mimics display lower *k*_obs_ compared to Mn1, confirming that they are more inert. In the case of Zn^2+^, Cu^2+^, and Co^2+^ exchanges, we observe the same ranking of increasing inertness between the SOD mimics, with the following order: Mn1, Mn1P, Mn1C, and lastly Mn1CP. These results are consistent with the previous UV-vis experiments. However, we observe a different trend for Ni^2+^ exchanges: Mn1P is surprisingly more inert than Mn1C, while Mn1P undergoes faster exchange than the other SOD mimics when in the presence of only one equivalent of Ni^2+^ according to UV-vis experiments. The stopped-flow experiments confirm the improved resistance to metal exchanges of the new SOD mimics and in particular of Mn1CP that displays the lower *k*_obs_ for each competitive metal ion exchange.

#### 3.2.2. Intrinsic SOD Activity

*(1) Electrochemistry*. For an efficient intrinsic SOD-like activity, redox potential of the SOD mimics has to be tuned between that of superoxide oxidation (*E*°′(O_2aq_/O_2_^−^) = −0.42 V/SCE, pH 7, 25°C) and reduction (*E*°′(O_2_^−^/H_2_O_2_) = 0.67 V/SCE, pH 7, 25°C) for an efficient catalysis of superoxide dismutation [74]. The electrochemical properties of the four Mn complexes were analyzed by cyclic voltammetry at a glassy carbon electrode in HEPES (50 mM, pH 7.5, ionic strength = 12.5 mM). The Mn^3+^/Mn^2+^ redox couples displayed reversible waves (see Figure S9) described in Table 1. The values obtained for Mn1 matched those previously reported (0.20 V/SCE) [44], and all of the complexes display a midpoint potential in the range 0.17-0.24 V/SCE, close to the optimal value for superoxide dismutation (0.12 V/SCE) [15]. It has been shown that the closer the Mn^3+^/Mn^2+^ midpoint redox potential to 0.12 V/SCE, the higher the intrinsic SOD activity (Figure S11) [15, 64, 75]. The inclusion of a cyclohexyl or a propyl group does not strongly impact the redox properties. We observe that the propylation of the secondary amine resulted in an increase in the midpoint potential. The propylation may induce a change in the complex geometry that disfavors Mn(III) [15]. A similar effect was observed elsewhere upon functionalization of the amine in enPI2 with cell-penetrating peptide (positively charged peptides) [43, 45].

*(2) McCord and Fridovich Assay*. The catalytic rate constants for superoxide dismutation *k*_cat_ of the studied SOD mimics were measured under conditions of slow flow of superoxide with the McCord-Fridovich assay in HEPES buffer (50 mM, pH 7.4). This assay is based on the competition between a SOD mimic and a visible probe specific for superoxide, XTT in this study, to react with superoxide [15, 46, 61, 62, 76]. This assay is relevant as a characterization of the kinetics under conditions of slow flow of superoxide, close to what is encountered in biological environment [15, 77]. In a series of complexes derived from Mn1, the McCord-Fridovich assay was shown to provide kinetic constants consistent to that obtained in a large excess of superoxide with stopped-flow [43, 45, 46] or pulse radiolysis [63]. In the McCord-Fridovich assay, the IC_50_ is defined as the concentration of SOD mimics that reduces by 50% of the reaction rate of the oxidation of XTT indicator (Figure S11). The *k*_cat_ of the four SOD mimics could be retrieved from their IC_50_ and *k*_XTT_ (2.9 × 10^4^ M^−1^ s^−1^) with *k*_cat_ = *k*_XTT_∗[XTT]/IC_50_ [15, 41, 64] and are reported in Table 1. The measured catalytic rate constants fall in a range already observed for similar complexes (≈10^7^ M^−1^ s^−1^) [63, 64]. We observed that the intrinsic SOD activity increased upon propylation of the secondary amine of EnPI2 and EnPI2C. This is consistent with results recently published by the group of E. Garcia-Espana. The authors reported an increase in SOD activity upon N-alkylation of tetra-azacyclophanes copper complexes [66]. They ran quantum mechanics/molecular mechanic molecular dynamic simulations to rationalize this effect. Briefly, in the absence of the alkyl groups on the secondary amines of the ligand (L), hydrogen bonds can be formed between O_2_^∙-^ and the hydrogen atoms belonging to the secondary amines, which results in an overstabilization of the catalyst-substrate adduct, O_2_^∙-^-Cu(II)-L. By increasing the energy barrier, this overstabilization slows down one-half reaction of superoxide dismutation: the reduction of the Cu(II) center to Cu(I) and the leaving of dioxygen. The addition of the alkyl group on the secondary amines allows to prevent the formation of these hydrogen bonds and thus increases the catalyst turnover. The intrinsic SOD activity also increased upon inclusion of a cyclohexyl group in EnPI2 structure. Mn1CP containing both the cyclohexyl and the propyl groups displayed the best intrinsic SOD activity. Lastly, we noticed that the *k*_cat_ results in the EnPI2 series are not consistent with the ranking associated with the electrochemical properties of the complexes. This suggests that other factors such as the kinetic inertness of the mimics or second coordination sphere effects associated with propylation (see above) may prevail over their weak differences in redox potentials [78].

### 3.3. Studies in LPS-Activated HT29-MD2 Cells

#### 3.3.1. Cellular Model

The four SOD mimics were assessed on a cellular model of inflammation mediated by oxidative stress. This model consists of intestinal epithelial cells named HT29-MD2, able to activate the inflammatory cascade NF*κ*B after recognition of bacterial lipopolysaccharide (LPS), a component of the bacterial cell membrane, by the Toll-like receptors (TLR-4) present at the cell surface. To increase their sensitivity to LPS, HT29 cells have been stably transfected to overexpress the MD2 protein, a soluble coreceptor of TLR-4 [59]. This model, in which the LPS-induced inflammatory reaction is associated with oxidative stress [44, 59, 79], was used to evaluate this bioactivity of various SOD mimics [41, 44–46, 48].

Prior to the biological evaluation, the cytotoxicity of the four mimics was evaluated by a LDH release assay (Figure S14). No cytotoxicity in HT29-MD2 cells (LDH released extracellularly by <10%) was observed for Mn1, Mn1P, and Mn1CP at 100 *μ*M when prepared with a 1 : 1 manganese-to-ligand ratio (6 h incubation). The noncoordinated ligands were found to be cytotoxic in the same conditions, as previously published in the case of EnPI2 [44]. The Zn(II) complexes were not cytotoxic: as redox-inactive complexes with the same overall charge as the Mn-SOD mimics, they constitute a relevant inactive control [44, 45]. A weak toxicity was observed in HT29-MD2 cells for Mn1C at 100 *μ*M prepared in the same conditions. This may be due to the slower and incomplete complexation of the cytotoxic EnPI2C ligand to manganese. To avoid that, all Mn complexes were prepared by mixing the ligand and MnCl_2_ in a 1 : 1.4 ratio for 2 hours prior to the cell incubation. Using this protocol, we achieved to reduce the extracellular LDH released below 10% even for Mn1C.

**Figure 4:**
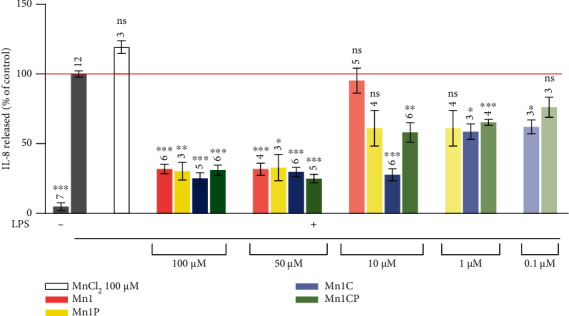
Quantification of the inflammatory marker IL-8 in intestinal epithelial cells activated with LPS (0.1 *μ*g/mL). IL-8 secretion was measured by ELISA in supernatant of LPS-activated HT29-MD2 cells incubated for 6 hours with the SOD mimics at different concentrations. The IL-8 amount measured for LPS-activated cells is set at 100% for each independent experiment. Data represent the mean ± SEM for at least three independent experiments: the number of independent experiments is indicated above each column. The *p* values were calculated using the Student *t*-test (bilateral test with equal variances not assumed). The mean ranks of each column were compared to that of the LPS control; each comparison stands alone. ^∗∗∗^*p* < 0.001, ^∗∗^*p* < 0.01, and ^∗^*p* < 0.05 versus LPS control, and ns means nonsignificant. Without LPS, no significant differences were observed between all of these conditions (see Figure S15). IL-8 secretions in control LPS+zinc complexes (Zn1, Zn1C, Zn1P, and Zn1CP) are shown in Figure S16.

**Figure 5:**
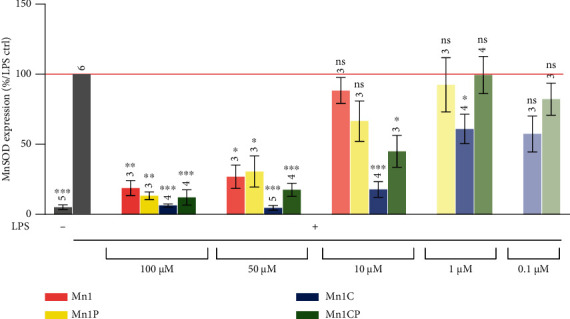
Quantification of MnSOD expression in intestinal epithelial cells activated with LPS (0.1 *μ*g/mL). MnSOD expression was measured by Western blot in lysates of LPS-activated HT29-MD2 cells incubated for 6 hours with the SOD mimics at different concentrations. The MnSOD expression intensity measured for activated cells is set at 100% for each independent experiment. The abundances of MnSOD were normalized to the total amount of protein in each lane. Data represent the mean ± SEM for at least three independent experiments: the number of independent experiments is indicated above each column. The *p* values were calculated using the Student *t*-test (bilateral test with equal variances not assumed). The mean rank of each column was compared to that of the LPS control; each comparison stands alone. ^∗∗∗^*p* < 0.001, ^∗∗^*p* < 0.01, and ^∗^*p* < 0.05 versus LPS control, and ns means nonsignificant. Without LPS, no significant differences were observed between all of these conditions (Figure S15). Control MnCl_2_ at 100 *μ*M was also assessed and did not display any antioxidant activity as expected (see Figure S13). Full blots are shown in Figure S12.

The following section summarizes the effect of the four SOD mimics in LPS-activated HT29-MD2 cells. For all experiments, HT29-MD2 cells were incubated with LPS at 0.1 *μ*g/mL to induce inflammation with or without the four SOD mimics at different concentrations for 6 hours. The anti-inflammatory and antioxidant activities of the SOD mimics were evaluated by looking at their ability to limit, respectively, the secretion of interleukin-8 (IL-8) and to reduce MnSOD overexpression induced by LPS (Figures 4 and 5) [41, 44–46, 48].

#### 3.3.2. Anti-Inflammatory Activity

As previously published, LPS activation induced a strong increase of IL-8 secretion in HT29-MD2 cell compared to control [41, 44, 45, 59]. When co-incubated in the presence of all of the studied SOD mimics at the non-toxic concentrations 50 *μ*M and 100 *μ*M, LPS-induced IL-8 levels were significantly reduced (<35% that of LPS positive control). The decrease of IL-8 secretion in response to LPS was not detected for Mn1 at 10 *μ*M, as previously published [45]. In contrast, Mn1P, Mn1C, and Mn1CP assayed at 10 *μ*M were still able to reduce the IL-8 production to 60%, 30%, and 50% that of LPS positive control, respectively, without reaching statistical significance for Mn1P and significantly for Mn1C and Mn1CP (Figure 4). Therefore, the new SOD mimics, particularly Mn1C, retain significant anti-inflammatory properties at lower doses than Mn1 (even at 1 *μ*M) and are thus efficacious against inflammation at lower incubation concentrations than Mn1. Non-significant effect on IL-8 secretion was observed for the corresponding zinc complexes Zn1, Zn1C, Zn1P, and Zn1CP at 10 *μ*M that constitutes relevant redox-inactive analogs of the manganese SOD mimics (Figure S16) [44, 45]. This confirms that these specific bioactivities are associated with the Mn complex redox properties. Zn1 activity on cells at 100 *μ*M was already controlled earlier [44] and reveals no effect on IL-8 secretion and MnSOD expression. Given the high kinetic rate of metal exchange from Mn1+Zn(II) to Zn1+Mn(II), Zn1 was the most important Zn analog to control at 100 *μ*M. Still, all the Zn analogs were evaluated at 10 *μ*M since at such concentrations about 30% of the SOD mimics should be theoretically dissociated (based on the association constants) and subjected to Zn coordination.

#### 3.3.3. Antioxidant Activity

The effect of the SOD mimics on MnSOD expression was investigated by Western blot analysis (Figure 5). As previously shown by Mathieu et al., the LPS activation of HT29-MD2 cells leads to a significant overexpression of catalytically active MnSOD. This is most probably related to the establishment of an oxidative stress situation associated with the appropriate cellular feedback response to counteract the LPS-induced oxidative stress [41, 44, 45]. The four studied SOD mimics limit significantly the LPS-induced MnSOD overexpression to 19%, 13%, 6%, and 12% that of LPS positive control, respectively, when incubated at 100 *μ*M. As previously reported, the SOD mimics seem to be able to complement for SOD in cells [41, 44, 45]. But at 10 *μ*M, the effect of Mn1 on MnSOD overexpression was almost completely suppressed (89% that of LPS positive control). In contrast, at 10 *μ*M, Mn1C and Mn1CP were still able to significantly limit MnSOD up-regulation (18% and 45% that of LPS positive control), whereas the activity of Mn1P was less efficient with an overexpression to 66% that of LPS positive control. This remanence of the anti-inflammatory and antioxidant activity of Mn1C and Mn1CP at concentrations lower than those required for Mn1 is consistent with the previous results showing the improved capacity of Mn1C and Mn1CP to fulfill the criteria required to get an efficient SOD mimic. Just as for IL-8 secretion, Mn1C can reduce MnSOD overexpression at lower doses than Mn1, Mn1P, and Mn1CP.

### 3.4. Intracellular Detection and Quantification

The detection and the quantification of the SOD mimics in HT29-MD2 lysates are important to support their involvement on the biological effects observed upon LPS stimulation.

**Figure 6:**
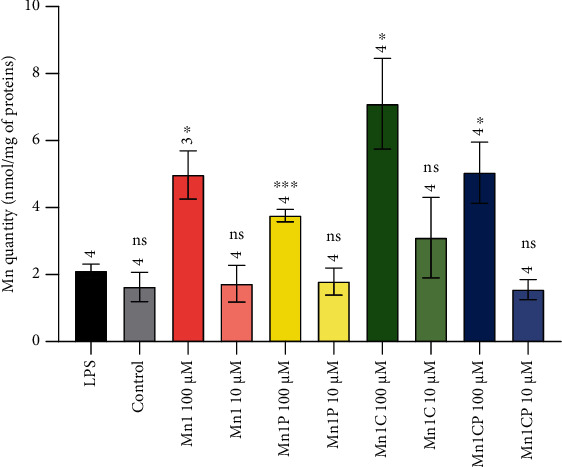
Quantification by ICP MS of total manganese content in LPS-activated HT29-MD2 cells incubated with the four SOD mimics at 10 and 100 *μ*M for 6 hours. Cell lysates were digested in HNO_3_ 2% to release manganese from all coordination sites. The amount of Mn in cell lysates was normalized by the mass of proteins determined by BCA assay. Data represent the mean ± SEM for at least three independent experiments: the number of independent experiments is indicated above each column. The *p* values were calculated using the Student *t*-test (bilateral test with equal variances not assumed). The mean rank of each column was compared to that of the LPS control; each comparison stands alone. ^∗∗∗^*p* < 0.001, ^∗∗^*p* < 0.01, and ^∗^*p* < 0.05 versus LPS control, and ns means nonsignificant. The *p* values and significance results corresponding to the comparison of the SOD mimics with each other are given in Figure S18.

The accumulation of the SOD mimics in HT29-MD2 cells was first checked by measuring the total amount of Mn content in cell lysates by ICP-MS (Figure 6). A significant increase from 2.1 (control LPS cells) to 6.4, 3.8, 7.1, and 5.0 nmol of Mn per mg of proteins was recorded in cells incubated with, respectively, Mn1, Mn1P, Mn1C, and Mn1CP at 100 *μ*M. This shows that the SOD mimics were all able to penetrate and accumulate significantly inside cells. At 100 *μ*M (incubation concentration), no significant difference in intracellular manganese amount was observed for any of the 4 SOD mimics. We only noticed that the addition of a propyl group whether in Mn1 or Mn1C structures results to a non-significant but systematic and visible decrease in the amount of Mn in cell lysate. This suggests a lower cell penetration of Mn1P and Mn1CP in comparison to their respective nonpropylated analogs Mn1 and Mn1C. This may contribute to the slightly improved bioactivity of Mn1C over Mn1CP in HT29-MD2 cells.

We then sought to detect the active species, i.e., intact manganese complexes, and to study their speciation in the cell lysates. To do so, we conducted mass spectrometry experiments on lysates of LPS-activated HT29-MD2 cells incubated with the SOD mimics at 100 *μ*M. In the case of Mn1, Mn1P, and Mn1C, the detection was biased either by deprotonation issues (Mn1 and Mn1C) happening in gas phase or by rapid metal exchanges with transition metals (Ni, Zn, and Fe) present endogenously or in the different parts of the analytical instrument (Figures S20, S21, S22, S23, S24, S25, S26, and S27). This prevented us either to detect them in the cell lysates or to determine accurately their cellular amount. However, the analysis of pure Mn1CP by mass spectrometry reveals that no metal exchange occurred between the Mn(II) center and the other metal ions present in the analytical system (Figure S26 and S27), making it suitable for unbiased quantification. Indeed, we observe two intense peaks at 504.24 and at 505.25 m/z corresponding to Mn1CP and its first isotope, while the peaks corresponding to the complex with other divalent metal cations are not visible except for Zn1CP but with a very weak intensity (20 times lower than that of Mn1CP).

**Figure 7:**
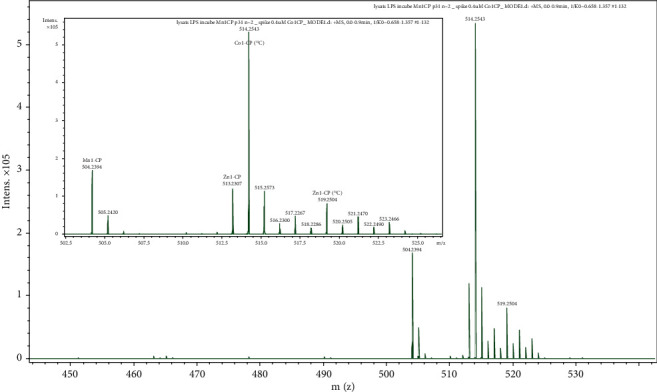
Mass spectrum of a LPS-stimulated HT29-MD2 lysate, previously incubated with the SOD mimic Mn1CP for 6 hours and ultracentrifuged at 100,000 rpm for 20 minutes. The lysate (10-15·10^6^cells in 2 mL NH_4_CO_3_, 50 mM) was diluted in 20% NH_4_CO_3_/80% ACN and spiked with the standard Co1CP (^13^C) at 0.4 *μ*M. The figure was zoomed onto the *m*/*z* region for the studied complexes. The peaks corresponding to Mn1CP, Co1CP (^13^C), and their isotopic pattern are clearly visible and annotated on the spectrum. Other similar mass spectra of cell lysates incubated with Mn1CP are given in Figure S30. Zn1CP(^13^C) displays peaks with relatively low intensity and was neglected for the quantification.

One example of mass spectrum corresponding to lysates of cells incubated with Mn1CP at 100 *μ*M is given in Figure 7. Three other examples of obtained mass spectra are given in Figure S30. We observed that Mn1CP partially dissociated and exchanged within the cell with Zn(II) to form mainly Zn1CP and, to a very small extent, Cu1CP. These exchanges were expected considering the high association constant of the ligand with Zn(II) (Figure S19). As we checked that the Zn(II) complexes do not have any activity in HT29-MD2 cells (Figure S16), this exchange merely led to a decrease in Mn1CP concentration in cells which is the bioactive molecule. We also checked the SOD activity of Cu complexes by McCord and Fridovich. Cu1, CuP1, Cu1C, and Cu1CP display SOD activity with the following catalytic rates: 0.34 × 10^6^ M^−1^ s^−1^, 0.63 × 10^6^ M^−1^ s^−1^, 0.60 × 10^6^ M^−1^ s^−1^, and 0.17 × 10^6^ M^−1^ s^−1^ (Figure S17). The measured catalytic rates are all slower than that of the copper salt (CuOAc)_2_ (14.510^6^ M^−1^ s^−1^). Then, the measured SOD activity could be due to released copper (Figure S17), to weakly active Cu complexes or to combination of both. Whatever the explanation, this again supports the conclusion that these exchanges solely result in a decrease of bioactive Mn1CP bioavailability and do not contribute to the observed activity in cells.

Mn1CP in lysates of HT29-MD2 cells was quantified by spiking a standard at 0.4 *μ*M before mass spectrometry analysis. The used standard consists of a heavy analog of Mn1CP, namely, Co1CP (^13^C) with a Co(II) ion center instead of Mn(II) and a ligand EnPI2CP bearing a phenol group labeled with ^13^C. ^13^C-labeled cobalt heavy complex constitutes a good standard as (a) it is very stable (*K*_1_Co1CP_ = 4.85 ± 1.15 × 10^8^ M^−1^, Figure S28), (b) it undergoes minimal metal exchange with the free divalent metal ions present in the cell lysates, and (c) it has physicochemical properties close to that of Mn1CP. The peaks corresponding to the standard (514.26 m/z, 515.26 m/z) are also clearly visible in Figure 7. In some cases, the spectra revealed relatively weak peaks corresponding to Zn1CP (^13^C) and Cu1CP (^13^C) that were neglected for the quantification. From the ratio of the signal intensity of Mn1CP with that of the standard Co1CP (^13^C) and using a calibration curve (Figure S29), the amount of Mn1CP in the cell lysates could be retrieved by taking into account the dilution done for the sample preparation for MS. Eventually, from the concentration of cells in the lysates, the intracellular molar amount of Mn1CP can be retrieved and was determined equal to 2.8 ± 0.5 × 10^−16^ mol per cells (mean ± SEM). The results of the quantification for the different cellular samples are detailed in Table S2 and were confirmed by the method of standard additions (Figure S31).

Owing the Irving-Williams series, or association constant, one could fear that no Mn(II) complex should be present in cells. However, as mentioned earlier, (i) the endogenous ligands that could compete with EnPI2-type ligands also have a weak affinity for Mn(II) (see above the association constant of the archetypal HSA for Mn(II)) and (ii) as highlighted by Lisher and Giedroc, metal ions such as Zn(II) or Cu(II) are indeed competitive metal ions, showing high association constants for most ligands, but their exchangeable pool is also very weak as they are tightly controlled in cells by proteins. In contrast, Mn(II) being a weaker binder is non-competitive but much more bioavailable [69]. This could explain that, despite the lower stability of Mn(II) complexes than Zn(II) or Cu(II) complexes, Mn1CP is clearly present in cells.

The presence of Mn1CP at 2.8 ± 0.5 × 10^−16^ mol per cells supports the accumulation and the persistence of the Mn complex inside the cells as well as its role in the decrease in IL-8 levels and in MnSOD expression observed in LPS-stimulated HT29-MD2.

## 4. Conclusion

The 1,2-diaminoethane central scaffold of the parent SOD mimic Mn1 was replaced by a (±)-trans-1,2-cyclohexyl group and substituted with a propyl group on its secondary amine in order to improve its kinetic inertness and to avoid any deprotonation during the speciation studies by mass spectrometry. Characterization of the resulting complexes demonstrated that these Mn1-derived SOD mimics displayed improved features for biological applications. Thanks to its rigidified skeleton, Mn1CP in particular is more resistant to metal exchanges that may lead to the formation of redox-inactive analogues in a biological medium. The modifications of the SOD mimics led to an improvement of the anti-inflammatory and antioxidant activities in a cellular model, consisting of epithelial intestinal cells HT29-MD2 under LPS-mediated inflammation and relevant to IBD. Indeed, Mn1C displays the best bioactivity, with significant effects when incubated at 10 *μ*M. These improved efficacies could be correlated to the higher bioavailability of the new SOD mimics in the cellular environment associated with a higher inertness. Note that, at this stage, we cannot exclude the possibility of a different subcellular location that could also be responsible for the better activity of Mn1C. This is now under exploration.

The rationalization of the SOD mimic bioactivities can be boosted by the direct detection and quantification of the active species inside the cells. The detection of open-chain manganese complexes in biological environments is not straightforward due to their high lability and thus possible metal exchanges. This is what is shown by the mass spectrometry experiments described in this work. For the most inert derivative Mn1CP, we investigate further its intracellular accumulation and speciation to quantify it in cells. Mn1CP partially exchanges Mn(II) with endogenous Zn(II) but remained present with an intracellular molar amount of 2.8 **×** 10^−16^ mol. The other Mn SOD mimics studied here were subjected to metal exchanges in the mass spectrometer and thus would require other metal-free techniques for unbiased detection.

The approach presented here, combining investigation of metal exchanges, bioactivity, and intracellular detection, indicates that kinetic inertness is a key property to be considered for biological applications. Overall, the newly designed more inert SOD mimics display a better ratio activity/concentration and thus constitute promising catalytic drug candidates for antioxidant and anti-inflammatory treatments in IBD context.

## Figures and Tables

**Table 1:** Association constants of the four ligands with manganese(II) (MnCl_2_) and the catalytic rate constant for superoxide dismutation observed with the four Mn complexes and their redox potentials.

L	*K*_1_ (Mn-L) (M^−1^)	*k*_cat_ (Mn-L) (10^6^ M^−1^ s^−1^)	*E*_cathodic_ (V/SCE)	*E*_anodic_ (V/SCE)	*E*_anodic_ − *E*_cathodic_ (mV)	*E*_1/2_ (V/SCE)
EnPI2	1.22 ± 0.18 × 10^6^	3.40 ± 0.10	0.16	0.24	80	0.20
EnPI2P	1.04 ± 0.18 × 10^6^	4.58 ± 0.01	0.17	0.29	120	0.23
EnPI2C	0.71 ± 0.11 × 10^6^	4.38 ± 0.13	0.12	0.21	90	0.17
EnPI2CP	0.28 ± 0.03 × 10^6^	5.41 ± 0.24	0.20	0.28	80	0.24

The association constants were determined by UV-vis titration experiments in HEPES (50 mM, pH 7.5, 25°C, C_ligand_ = 45 *μ*M; see Figure S2) and are indicated with the 95% confidence intervals based on asymptomatic standard errors. These values were calculated using the MATLAB curve fitting tool based on a nonlinear least squares regression method. The calculation of *K*_1_ and the UV titrations plots are detailed in supplementary information and in Figure S2. The catalytic rate constants for superoxide dismutation of the manganese complexes were measured in HEPES (50 mM at pH 7.4) using the McCord and Fridovich assay (Figure S11). The anodic, cathodic, Δ*E*, and midpoint potentials (*E*_1/2_ = (*E*_c_ + *E*_a_)/2) of the four manganese complexes were extracted from their cyclic voltammogram given in Figure S9.

**Table 2:** Half-lives of the metal-assisted dissociation reaction of manganese complexes.

		Zn^2+^	Cu^2+^	Ni^2+^	Co^2+^
*t*_1/2_ (s)	Mn1	n.d	4	45	11
	Mn1-P	n.d	17	16	16
	Mn1-C	22	28	120	50
	Mn1-CP	188	139	133	60

The exchanges were monitored by UV-visible spectrophotometry after addition of one equivalent of salts of the exchanging metal ions (ZnCl_2_, CuSO_4_, NiCl_2_·6H_2_O, or CoCl_2_) in a solution of the manganese complexes at 100 *μ*M (Tris 50 mM, pH 7.5). For Zn^2+^, Cu^2+^, and Co^2+^, the experiments were performed at 5°C as the metal exchanges were too fast at room temperature (25°C) preventing any kinetic study. For Ni^2+^ exchanges study, the temperature was maintained at 25°C. n.d means not determined; as in some cases, the exchanges were too fast, even at low temperature, and could not be monitored by UV-vis spectroscopy.

## Data Availability

The data presented in this study are given in supplementary figures and information.

## Supplementary Materials

Supplementary Materials Material and reagents section, instrumentation section, protocol for the synthesis of the four ligands EnPI2/EnPI2P/EnPI2C/EnPI2CP and the ^13^C labeled EnPI2CP ligand, and calculation used for *K*_1_ determination from basic UV titrations and from competition experiments. Supplementary table: pseudo-first-order rate constants *k*_obs_ and half-lives of the metal exchanges between the Mn(II) center and Zn(II), Ni(II), Cu(II), and Co(II) for each SOD mimic (Table S1) and quantification values of Mn1CP amount in cell lysates (Table S2). Supplementary figure: structure of EDTA, CDTA, PyC3A, and ligands mentioned in this study (Figure S1), determination of the association of Mn(II), Zn(II), and Co(II) complexes (Figures S2, S19, and S28), UV-vis spectra of Mn(II), Zn(II), Ni(II), Cu(II), and Co(II) complexes (Figures S3–S6), kinetic study of the metal exchanges between the Mn(II) center and Zn(II), Ni(II), Cu(II), and Co(II) for each SOD mimic (Figure S7), HPLC chromatograms of the ligands (Figure S8), cyclic voltammograms of the studied SOD mimics (Figure S9), structure of other Mn(II) complexes bioinspired from SOD (Figure S10), McCord and Fridovich assay data for SOD mimics (Figure S11), Western blot results/blots and IL-8 results for each SOD mimic (Figure S12), Western blot for MnCl_2_ at 100 *μ*M control (Figure S13), LDH assay results (Figure S14), IL-8 and MnSOD data for controls without LPS (Figure S15), IL-8 and LDH assay data for Zn(II) complexes controls (Figure S16), McCord and Fridovich assay data for Cu complexes and free Cu^2+^ (Figure S17), statistical results of intracellular Mn quantification by ICP MS (Figure S18), TOF-MS spectra of Mn(II) complexes (Figures S20–S27), calibration curve for quantification of Mn1CP (Figure S29), TOF-MS spectra of lysates of HT29-MD2 cells incubated with Mn1CP at 100 *μ*M (Figure S30), and addition standard method for Mn1CP quantification in cells (Figure S31).
