# Supplementary material for: Inertness of Superoxide Dismutase Mimics Mn(II) Complexes Based on an Open-Chain Ligand, Bioactivity, and Detection in Intestinal Epithelial Cells
Source: Oxid Med Cell Longev. 2022 Apr 1;2022:3858122. doi: 10.1155/2022/3858122 (PMC8993562; doi:10.1155/2022/3858122)
Supplement: Supplementary Materials — Material and reagents section, instrumentation section, protocol for the synthesis of the four ligands EnPI2/EnPI2P/EnPI2C/EnPI2CP and the 13C labeled EnPI2CP ligand, and calculation used for K1 determination from basic UV titrations and from competition experiments. Supplementary table: pseudo-first-order rate constants kobs and half-lives of the metal exchanges between the Mn(II) center and Zn(II), Ni(II), Cu(II), and Co(II) for each SOD mimic (Table S1) and quantification values of Mn1CP amount in cell lysates (Table S2). Supplementary figure: structure of EDTA, CDTA, PyC3A, and ligands mentioned in this study (Figure S1), determination of the association of Mn(II), Zn(II), and Co(II) complexes (Figures S2, S19, and S28), UV-vis spectra of Mn(II), Zn(II), Ni(II), Cu(II), and Co(II) complexes (Figures S3–S6), kinetic study of the metal exchanges between the Mn(II) center and Zn(II), Ni(II), Cu(II), and Co(II) for each SOD mimic (Figure S7), HPLC chromatograms of the ligands (Figure S8), cyclic voltammograms of the studied SOD mimics (Figure S9), structure of other Mn(II) complexes bioinspired from SOD (Figure S10), McCord and Fridovich assay data for SOD mimics (Figure S11), Western blot results/blots and IL-8 results for each SOD mimic (Figure S12), Western blot for MnCl2 at 100 μM control (Figure S13), LDH assay results (Figure S14), IL-8 and MnSOD data for controls without LPS (Figure S15), IL-8 and LDH assay data for Zn(II) complexes controls (Figure S16), McCord and Fridovich assay data for Cu complexes and free Cu2+ (Figure S17), statistical results of intracellular Mn quantification by ICP MS (Figure S18), TOF-MS spectra of Mn(II) complexes (Figures S20–S27), calibration curve for quantification of Mn1CP (Figure S29), TOF-MS spectra of lysates of HT29-MD2 cells incubated with Mn1CP at 100 μM (Figure S30), and addition standard method for Mn1CP quantification in cells (Figure S31). [file 3858122.f1.docx]

Table of contents

[Supplementary information 2](#_Toc90894649)

[Material and reagents 2](#_Toc90894650)

[Instrumentation 3](#_Toc90894651)

[Synthesis of EnPI2 3](#_Toc90894652)

[Synthesis of EnPI2P 4](#_Toc90894653)

[Synthesis of EnPI2C 4](#_Toc90894654)

[Synthesis of EnpI2CP 6](#_Toc90894655)

[Synthesis of EnpI2CP labelled with ^13^C 7](#_Toc90894656)

[K_1_ calculation of MnL complexes from UV titration experiments 8](#_Toc90894657)

[K_1_ calculation of ZnL/Co1CP complexes: competition with the formation of MnL 9](#_Toc90894658)

[Supplementary tables 10](#_Toc90894659)

[Table S1: (a) Pseudo-first order rate constants k_obs_ (s^-1^) and (b) half-lives (s) characterizing the metal exchanges occurring between the Mn^2+^ center of the SOD mimics with Zn^2+^, Cu^2+^, Ni^2+^ and Co^2+^ 11](#_Toc90894660)

[Table S2: Quantification of Mn1CP in the lysates of HT29-MD2 cells 11](#_Toc90894661)

[Supplementary figures 11](#_Toc90894662)

[Figure S1: (A) Structure of EDTA (ethylenediamine tetraacetic acid), (B) Structure of CDTA (cyclohexanediamine tetraacetic acid), (C) Structure of PyC3A, (D) Structure of EnPI2, (E) Structure of EnPI2P, (F) Structure of EnPI2C and (G) Structure of EnPI2CP. 11](#_Toc90894663)

[Figure S2: Determination of the association constants K_1_ of the studied SOD mimics from UV-titration experiments. 12](#_Toc90894664)

[Figure S3: UV-visible spectra of (A) Mn1 and the Zn(II) complex Zn-EnPI2 labelled Zn1, (B) Mn1P and the Zn(II) complex Zn-EnPI2P labelled Zn1P, (C) Mn1C and the Zn(II) complex Zn-EnPI2C labelled Zn1C and (D) Mn1CP and the Zn(II) complex Zn-EnPI2CP labelled Zn1CP. 13](#_Toc90894665)

[Figure S4: UV-visible spectra of (A) Mn1 and the Cu(II) complex Cu-EnPI2 labelled Cu1, (B) Mn1P and the Cu(II) complex Cu-EnPI2P labelled Cu1P, (C) Mn1C and the Cu(II) complex Cu-EnPI2C labelled Cu1C and (D) Mn1CP and the Cu(II) complex Cu-EnPI2CP labelled Cu1CP. 13](#_Toc90894666)

[Figure S5: UV-visible spectra of (A) Mn1 and the Ni(II) complex Ni-EnPI2 labelled Ni1, (B) Mn1P and the Ni(II) complex Ni-EnPI2P labelled Ni1P, (C) Mn1C and the Ni(II) complex Ni-EnPI2C labelled Ni1C and (D) Mn1CP and the Ni(II) complex Ni-EnPI2CP labelled Ni1CP. 13](#_Toc90894667)

[Figure S6: UV-visible spectra of (A) Mn1 and the Co(II) complex Co-EnPI2 labelled Co1, (B) Mn1P and the Co(II) complex Co-EnPI2P labelled Co1P, (C) Mn1C and the Co(II) complex Co-EnPI2C labelled Co1C and (D) Mn1CP and the Co(II) complex Co-EnPI2CP labelled Co1CP. 14](#_Toc90894668)

[Figure S7: UV-vis kinetics study of the metal exchanges occurring between the manganese center of the SOD mimics and respectively (A) Zn^2+^ ions, (B) Cu^2+^ ions, (C) Ni^2+^ ions and (D) Co^2+^ ions 15](#_Toc90894669)

[Figure S8: HPLC analysis of the four EnPI2-derivated ligands on a C_18_ column 16](#_Toc90894670)

[Figure S9: Cyclic voltammograms of the SOD mimics 17](#_Toc90894671)

[Figure S10: Mn(II)-complexes bio-inspired from SOD. 17](#_Toc90894672)

[Figure S11: McCord and Fridovich assays of SOD mimics performed at pH 7.4 in HEPES 50 mM with the use of XTT as redox competitive indicator. 18](#_Toc90894673)

[Figure S12: Evaluation of the antioxidant and anti-inflammatory activity of Mn1, Mn1C, Mn1P and Mn1CP in intestinal epithelial cells HT29-MD2 activated with LPS (0.1 μg/mL) 19](#_Toc90894674)

[Figure S13: Evaluation of the antioxidant activity of MnCl_2_ incubated at 100 µM in intestinal epithelial cells HT29-MD2 activated with LPS (0.1 μg/mL). 20](#_Toc90894675)

[Figure S14: Evaluation of the cytotoxicity induced by the SOD mimics using the LDH assay. 20](#_Toc90894676)

[Figure S15: Quantification of IL-8 secretion and of MnSOD expression in controls without LPS. 21](#_Toc90894677)

[Figure S16: Evaluation of the anti-inflammatory activity and of the cytotoxicity of the four zinc complexes in intestinal epithelial cells activated with LPS (0.1 μg/mL) 22](#_Toc90894678)

[Figure S17: McCord and Fridovich assays of Cu-complexes and free Cu^2+^ performed at pH 7.4 in HEPES 50 mM with the use of XTT as redox competitive indicator. 22](#_Toc90894679)

[Figure S18: Statistical results of manganese quantification by ICP-MS in LPS-activated HT29-MD2 cells incubated with the 4 studied SOD mimics at 10µM and 100µM 23](#_Toc90894680)

[Figure S19: Determination of the association constants K_1_ of the studied ligands with Zn(II) from competition experiments in HEPES 50 mM pH 7.5. 23](#_Toc90894681)

[Figure S20: Mass spectrum of Mn1 diluted at 0.2 µM in 20% NH_4_CO_3_/ 80% ACN 24](#_Toc90894682)

[Figure S21: Predicted isotopic patterns of Mn1 (left) and Zn1 (right). 24](#_Toc90894683)

[Figure S22: Mass spectrum of Mn1P diluted at 2 µM in 20% NH_4_CO_3_/ 80% ACN. 25](#_Toc90894684)

[Figure S23: Predicted isotopic patterns of Mn1P (left) and Zn1P (right). 25](#_Toc90894685)

[Figure S24: Mass spectrum of Mn1C diluted at 0.2 µM in 20% NH_4_CO_3_/ 80% ACN 26](#_Toc90894686)

[Figure S25: Predicted isotopic patterns of Mn1C (left) and Zn1C (right). 26](#_Toc90894687)

[Figure S26: Mass spectrum of Mn1CP diluted at 0.2 µM in 20% NH_4_CO_3_/ 80% ACN. 27](#_Toc90894688)

[Figure S27: Predicted isotopic patterns of Mn1CP (left) and Zn1CP (right). 27](#_Toc90894689)

[Figure S28: Determination of the association constants K_1_ of EnpI2CP ligand with Co(II) from competition experiments with Mn1CP 27](#_Toc90894690)

[Figure S29: Calibration curves for Mn1CP quantification obtained in cell lysates diluted in 20% NH_4_CO_3_/ 80% ACN 28](#_Toc90894691)

[Figure S30: Examples of mass spectra of a LPS-stimulated HT29-MD2 lysates, previously incubated with the SOD mimic Mn1CP for 6 hours 30](#_Toc90894692)

[Figure S31: Quantification of Mn1CP in LPS-stimulated HT29-MD2 lysates by the method of standard additions. 30](#_Toc90894693)

[Abbreviations 30](#_Toc90894694)

# Supplementary information

## Material and reagents

Chemicals and solvents were purchased from commercial sources (Sigma-Aldrich, Alfa-Aesa, Strem, Acros, Iris) and were used as received without further purification.

For ICP-MS, nitric acid (HNO_3_) 65% Suprapur was purchased from Merck (#1.00441.1000). Manganese Standard for ICP (1003 mg/L ± 4 mg/L, TraceCERT #74128) were from Sigma Aldrich.

For MS detection of Mn1-CP inside cells, ultrapure ULC/MS – CC/SFC grade solvents were purchased from Biosolve: 2-propanol (#162641), Acetonitrile (#12041), Water (#232141). Syringes for IMS-MS direct infusion were purchased from Agilent: Manual syringe, removable needle 22/51/LC, PTFE-tip plunger 500 μL (#5190-1526), 250 μL (#5190-1520), 100 μL (#5190-1512). PFA (metal-free) labware were purchased from AHF ANALYSENTECHNIK: Narrow Neck Bottle 100 mL (T81-100), Micro Spatulas made of CTFE (T29-578), Measuring Cylinder 50 mL (T89-050).

LPS (Escherichia coli O55:B5), NADH, pyruvic acid, BSA, Tween 20 and Triton 100X were purchased from Sigma Aldrich (Saint-Quentin Fallavier, France). IL8 detection ELISA kit (Duoset) was provided by R&D Systems (Minneapolis, Minnesota, USA). Horseradish peroxidase-conjugated antibodies were from Jackson ImmunoResearch (Ely, UK). BCA was from Uptima-Interchim (Montluçon, France). Detection ECL system, protein gels (Mini Protean TGX Stain free Precast Gel) and nitrocellulose membranes were from Biorad. Blasticidin were purchased from Invivogen (Toulouse, France). Rabbit polyclonal anti-human SOD 2 antibodies were from Invitrogen (Thermo Fisher Scientific, Waltham, Massachussetts, USA). Fetal bovine serum was from GE Healthcare Life Sciences (South Logan, Utah, USA). Dulbecco’s modified Eagle medium (DMEM), HEPES buffer solution (1 M), 0.05% trypsin-EDTA and Dulbecco’s Phosphate Buffered Saline (10X, DPBS) was from Gibco (Thermo Fisher Scientific, Waltham, Massachussetts, USA). The protease inhibitor cocktail was from Roche Diagnostics (reference: 11836145001, Sigma-Aldrich, Meylan, France). The mass and IMS calibration of the timsTOF was done with continuous infusion of ESI-Low Concentration Tuning Mix from Agilent (Santa Clara, USA).

## Instrumentation

UV-vis spectra were recorded on a Cary 300 spectrophotometer (Agilent technologies, Santa Clara, CA, USA) using a double-beam mode with media as the reference. Plastic UV-cuvettes, semi-micro (1.5 mL) were purchased from BRAND (12.5 x 12.5 x 45 mm, #7591 50). UV−visible spectra (for all biological experiments) were recorded on a SpectraMax M5 Series Multi-Mode Microplate Reader from Molecular Devices (CRSA, Sorbonne University Medicine campus, with the courtesy of Dr. Philippe Seksik). Analytical HPLC was performed on an Agilent 1200 series equipped with a quaternary pump using a Proto 200 C18 from Higgins Analytical Inc (particles size 3 μm, 100×4.6 mm column). Preparative HLC was performed on an Agilent 1260 Infinity using a Nucleodur C18 HTech column from Macherey-Nagel Inc. (particles size 5 μm, 250×16 mm column). ICP-MS analyses were performed on an Agilent 7700 X. ^55^Mn were selected as isotopes to avoid isobaric interferences. ESI-MS experiments (HRMS) were carried out using a LTQ-Orbitrap XL from Thermo Scientific (Thermo Fisher Scientific, Courtaboeuf, France) and operated in positive ionization mode, with a spray voltage at 3.6 kV. Applied voltages were 20 and 70 V for the ion transfer capillary and the tube lens, respectively. The ion transfer capillary was held at 275°C. Detection was achieved in the Orbitrap with a resolution set to 100,000 (at m/z 400) and a m/z range between 190-1500 in profile mode. Spectrum was analyzed using the acquisition software XCalibur 2.1 (Thermo Fisher Scientific, Courtaboeuf, France). The automatic gain control (AGC) allowed accumulation of up to 2.10^5^ ions for FTMS scans, Maximum injection time was set to 300 ms and 1 µscan was acquired. 10 µL was injected using a Thermo Finnigan Surveyor HPLC system (Thermo Fisher Scientific, Courtaboeuf, France) with a continuous infusion of methanol at 100 µL.min^-1^. ^1^H and ^13^C NMR spectra were recorded on a Bruker DRX300 Ultrashield model spectrometer with a proton and carbon frequency of 300 MHz and 75 MHz respectively. Spectra have been analyzed using the software MestReNova 7.1. The chemical shifts (δ/ppm) were calibrated relative to residual solvent signals. The following abbreviations are used: singlet (s), doublet (d), triplet (t) and multiplet (m). MS characterization of ligands were conducted with classic electrospray (ESI) 4 kV source (Thermo Fisher Scientific) in positive mode coupled to a Qq Orbitrap mass spectrometer; Q Exactive Hybrid Quadrupole-Orbitrap from Thermo Fisher Scientific (SMBP, ESPCI, with the courtesy of Dr. Joelle Vinh). Direct infusion was performed at 5 µL/min. The ligands (EnPI2P, EnPI2C, EnPI2CP) were analyzed at 100 μM in 85% ACN/ammonium formate 50 mM at pH 6.5. For cyclic voltammetry, a MetrOhm potentiostat (AUTOLAB model) was used. The auxiliary electrode was a Pt wire and the working electrode was a glassy carbon disk (3 mm diameter) carefully polished before each voltammogram with a 1 μm diamond paste, washed with water and finally air dried. The reference electrode was a SCE saturated with KCl. For Mn1CP detection and quantification in cell lysates, the TOF-MS part of the timsTOF provided by Bruker Daltonics (Champ sur Marne, France) were used (IPREM-UMR5254, Pau France). The experiment operated in positive ionization mode and tims detect mode (IMS resolution 120), with a capillary voltage at 4.5 kV. The dry gas transfer capillary was held at 230°C. Detection was achieved in the timsTOF with a resolution set to 70,000 (at m/z 1221) and a m/z range between 200-1250 in profile focused mode. Spectrum was analyzed using the acquisition software DataAnalysis (Bruker Daltonics, Champ sur Marne, France). The acquisition frequency was set to 2 Hz. The sample was first injected during 1 minute for stabilization and 1 additional minute for acquisition, using the embedded infusion pump system from the timsTOF with a continuous infusion flow of 180 µL/min. Stopped-flow experiments were performed on a SFM-3000/S stopped-flow mixer equipped with a HDS mixer and kelraz o-ring, all provided by BioLogic (Seyssinet-Pariset, France). The stopped-flow mixer was connected to a control unit MPS-70/3 (Biologic), a deuterium/tungsten light source 30W (BioLogic) and a MMS-UV/vis-ASPEN- high speed diode array spectrometer. The stopped-flow experiments were controlled using the software Biokine32 (BioLogic).

## Synthesis of EnPI2

EnPI2 was synthesized according to a previously reported procedure [1].

## Synthesis of EnPI2P

Propionaldehyde (960 µL, 13.31 mmol, 1.5 equiv.) was added to a solution of EnPI2 (3.15 g, 8.87 mmol) in absolute ethanol (30 mL). After stirring the reaction mixture for 2 h at room temperature under argon, NaBH_3_CN (0.558 g, 8.87 mmol, 1 equiv.) was added and pH was adjusted to 5 using NaOH 1 M or HCl 1 M. The reaction mixture was stirred two additional hours at room temperature. A 1 M NaOH solution was then added until pH 7 and EtOH was evaporated. The product was resuspended in H_2_O (20 mL) and dichloromethane (DCM) (20 mL) and pH was adjusted to 9. The aqueous phase was extracted twice with DCM (2 × 20 mL) and the organic phase was dried over anhydrous sodium sulfate. DCM was eventually evaporated to give crude product. The product was purified by preparative HPLC using an ACN/H_2_O (with 0.1 % TFA) gradient going from 10/90 to 40/60 in 30 min. After solvent evaporation, a yellow oil was obtained with a yield of 31%.

^1^H NMR (EnPI2P) (300 MHz, CD_3_OD): δ = 7.7 (m, 2 H, H_Ar_), 7.5 (s,2 H, H_Ar_), 7.3 (m, 1 H, H_Ar_), 7.2 (t, J=6Hz, 1H, H_Ar_), 6.9 (m, 2 H, H_Ar_), 4.9 (s, 2 H, N-C**H_2_**-C_Im_), 4.5 (s, 2 H, N-C**H_2_**-C_Im_), 4.1 (s, 5 H, NC**H_3_** + N-C**H_2_**-C_Ph_), 3.8 (s, 3 H, NC**H_3_**), 3.6 (t, J=6 Hz, 2 H, N-C**H_2_**-C**H_2_**-N), 3.4 (t, J=6 Hz, 2 H, N-C**H_2_**-CH_2_-N), 3.1 (m, 2 H, N-CH_2_-C**H_2_**-CH_3_), 1.8 (m, 2 H, N-C**H_2_**-C**H_2_**-CH_3_), 1.0 (t, J=7.5Hz, 3 H, N-CH_2_-CH_2_-C**H_3_**).

^13^C NMR (EnPI2P) (75 MHz, CD_3_OD): δ = 156.8 (C_Ph_-OH), 146.0 (C_quat,Im_), 144.8 (C_quat,Im_), 132.3 (C_Ph_-H), 130.5 (C_Ph_-H), 125.2 (C_Im_H), 124.9 (C_Im_H), 123.4 (C_quat,Ph_), 121.5 (C_Ph_-H), 120.6 (C_Im_H), 119.6 (C_Im_H), 116.2 (C_Ph_-H), 57.5 (N-**C**H_2_-CH_2_-CH_3_), 55.4 (C_Ph_-**C**H_2_), 52.8 (N-**C**H_2_-**C**H_2_-N), 52.6 (N-**C**H_2_-**C**H_2_-N), 49.6 (N-**C**H_2_-C_Im_), 49.1 (N-**C**H_2_-C_Im_), 34.6 (N-**C**H_3_), 34.5 (N-**C**H_3_), 19.8 (N-CH_2_-**C**H_2_-CH_3_) and 11.5 (N-**C**H_2_-CH_2_-**C**H_3_) ppm.

HRMS (ESI) m/z: [M+H]^+^ calculd for C_22_H_32_N_6_OH 397.2710. Found 397.2712; (Error: 0.4 ppm).

Single isotopic mass : 397.3 m/z.

## Synthesis of EnPI2C

EnPI2C was synthesized according to a previously reported procedure [2] using the racemic (±)-(trans)-1,2-diamino cyclohexane instead of 1,2-diaminoethane as reagent.

P1 (0.5 g, 4.54 mmol, 2 equiv.) was dissolved in ACN (0.5 mL). (±)-(trans)-1,2-diamino cyclohexane (273 µL, 2.27 mmol, 2 equiv.) was then added and the reaction mixture was stirred under argon until precipitation ended. The precipitate was then filtered off and washed with petrol ether to afford a yellow solid with a yield of 92%.

^1^H NMR (P2) (300 MHz, CDCl_3_): δ = 8.2 (s, 2 H, N=CH-Im), 7.0 (d, J=3 Hz, 2 H, CH_Im_), 6.9 (d, J=3 Hz, 2 H, CH_Im_), 3.9 (s, 6 H, NCH_3_), 3.3 (bm, 2 H, CH_cyclo_), 1.8 (m, 6 H, CH_cyclo_), 1.5 (m, 2 H, CH_cyclo_).

^13^C NMR (P2) (75 MHz, CDCl_3_): δ = 152.2 (N=**C**H-Im, 2 C), 143.2 (C_quatIm_, 2 C), 128.9 (C_Im_H, 2 C), 124.9 (C_Im_H, 2 C), 74.8 (N-CH_cyclo_, 2 C), 42.9 (**C**H_2,cyclo_, 2 C), 35.4 (N-**C**H_3_, 2 C), 32.9 (**C**H_2,cyclo_, 2 C), 24.3 (**C**H_2,cyclo_, 2 C)

P2 (0.621 g, 2.08 mmol) was dissolved in absolute ethanol (10 mL) and NaBH_4_ (0.236 g, 6.25 mmol, 3 equiv.) was added. After two hours of stirring at room temperature under argon, HCl 1 M was added to the reaction to pH 7. Water (10 mL) and DCM (20 mL) were added and p5 was extracted in the DCM fraction. The extraction was repeated twice. P3 was then dried over Na_2_SO_4_, filtered off and DCM was evaporated to afford a yellow solid with a yield of 90%.

^1^H NMR (P3) (300 MHz, CDCl_3_): δ = 6.8 (d, J=3 Hz, 2 H, N-CH=CH), 6.7 (d, J=3 Hz, 2H, N-CH=CH), 3.9-3.6 (bm, 4 H, N-CH_2_-Imi), 3.6 (s, 6 H, N-CH_3_), 2.2-2.1 (bm, 4 H, CH_cyclo_), 1.7 (m, 2 H, CH_cyclo_), 1.2-1.0 (bm, 4 H, CH_cyclo_)

^13^C NMR (P3) (CDCl_3_, 75 MHz): δ = 146.7 (C_quat,Im_, 2 C), 126.7 (C_Im_H, 2 C), 121.1 (C_Im_H, 2 C), 61.0 (N-CH_cyclo_, 2 C), 42.9 (N-CH_2_-Im, 2 C), 32.8 (N-**C**H_3_, 2 C), 31.2 (**C**H_2,cyclo_, 2 C), 24.2 (**C**H_2,cyclo_, 2 C)

P3 (0.64 g, 2.12 mmol) was dissolved in absolute ethanol (10 mL). Salicylaldehyde (223 µL, 2.12 mmol, 1 equiv.) was added and the reaction mixture was stirred for 48 hours under argon at room temperature. The reaction mixture was then dried over anhydrous sodium sulfate (Na_2_SO_4_) and filtered off under vacuum. Ethanol was removed by rotary evaporation under vacuum to afford P4 with a quantitative yield.

^1^H NMR (P4) (300 MHz, CDCl_3_): δ = 7.1-6.4 (bm, 8 H, H_Ar_), 3.8 (m, 2 H, N-CH_2_-Ph), 3.6-3.5 (m, 2 H, N-CH_2_-Im), 3.4 (s, 3 H, N-CH_3_), 3.2 (s, 3 H, N-CH_3_), 2.8 (m, 1 H, N-CH-CH-N), 2.5 (m, 1 H, N-CH-CH-N), 1.7-1.5 (bm, 3 H, CH_cyclo_), 1.3-0.8 (bm, 5 H, CH_cyclo_)

^13^C NMR (P4) (CDCl_3_, 75 MHz): δ = 157.5 (C_Ph_-OH), 145.4-143.3 (C_quat,Im_, 2 C), 130.7-129.6 (C_Ph_-H, 2 C), 127.0-126.4 (C_Im_H, 2 C), 122.0 (C_quat,Ph_), 121.5-121.1 (C_Im_H, 2 C),119.0-116.4 (bm, C_Ph_-H, 2 C), 86.7 (N-CH-N), 68.2-67.8 (N-CH-CH-N, CH_cyclo_, 2 C) , 47.6-45.7 (N-CH_2_-Im, 2 C), 32.8-32.5 (N-CH_3_, 2 C), 29.2 (CH_cyclo_, 2 C), 24,2 (CH_cyclo_, 2 C)

P4 (0.906 g, 2.22 mmol) was dissolved in of absolute ethanol (20 mL). Sodium cyanoborohydride NaBH3CN (0.139 g, 2.22 mmol, 1 equiv.) and trifluoroacetic acid TFA (171 μL, 2.22 mmol, 1 equiv.) were added and the reaction mixture was stirred for 2 hours at room temperature. A color change from yellow to colorless was observed. The pH was then adjusted to 8-8.4 by adding NaOH 1 M to neutralize the TFA. After removing the ethanol by rotary evaporation, DCM (20 mL) and water (20 mL) were added. The aqueous layers were then extracted with DCM (3 x 20 mL). The extraction was monitored by measuring the absorbance at 254 nm (one droplet was deposited on a silica gel TLC plate). The combined organic layers were dried over anhydrous sodium sulfate and filtered off. DCM was removed by rotary evaporation and the product was purified by preparative HPLC using a ACN/H_2_O (with 0.1 % TFA) gradient going from 10/90 to 30/70 in 30 minutes. After solvent evaporation, we afford a yellow powder (1.36 g, 3.33 mmol) with a yield of 36%.

^1^H NMR (EnPI2C) (300 MHz, CD_3_OD): δ = 7.7 (m, 2 H, H_Ar_), 7.2 (m, 3 H, H_Ar_), 7.1 (m, 1 H, H_Ar_), 6.82 (m, 2 H, H_Ar_), 4.2 (m, 2 H, N-C**H_2_**-Im), 4.1 (m, 5 H, NC**H_3_** + N-C**H_2_**-Im), 3.9 (m, 2 H, N-C**H_2_**-Ph), 3.61 (s, 3 H, N-C**H_3_**), 3.5 (m, 1 H, N-C**H**_cylo_-C**H**_cyclo_-N), 3.2 (m, 1 H, N-C**H**_cylo_-C**H**_cyclo_-N), 2.4 (m, 1 H, C**H**_2,cyclo_), 2.2 (m, 1 H, C**H**_2,cyclo_), 1.9 (m, 2 H, C**H**_2,cyclo_), 1.7-1.4 (bm, 4 H, C**H**_2,cyclo_).

^13^C NMR (EnPI2C) (75 MHz, D_2_O): δ = 153.9 (C_Ph_-OH), 143.4 (C_quat,Im_), 136.5 (C_quat,Im_), 131.3 (C_Ph_-H), 130.1 (C_Ph_-H), 125.6 (C_Im_H), 123.4 (C_Im_H), 122.5 (C_quatPh_), 121.0 (C_Ph_-H), 120.6 (C_Im_H), 118.2 (C_Im_H), 115.8 (C_Ph_-H), 65.1-60.5 (N-**C**H_cylo_-**C**H_cyclo_-N), 49.8 (N-**C**H_2_-Ph), 46.3 (N-**C**H_2_-Im), 36.7 (N-**C**H_2_-Im), 35.1 (N-**C**H_3_, 1 C), 33.8 (N-**C**H_3_, 1 C), 27.1-24.5-23.7-22.7 (**C**H_2_,_cyclo_, 4 C)

HRMS (ESI) m/z: [M+H]+ Calcd for C_23_H_32_N_6_OH 409.2710. Found 409.2712; (Error: 0.4 ppm).

Single isotopic mass : 409.3 m/z.

## Synthesis of EnpI2CP

The propylation of EnPI2C to obtain EnPI2CP is performed similarly to the propylation of EnPI2 to obtain EnPI2P (see above).

Propionaldehyde (1160 µL, 16.12 mmol, 2.5 equiv.) was added to a solution of EnPI2C (2.635 g, 6.45 mmol) in absolute ethanol (50 mL). After stirring the reaction mixture for 2 h at room temperature under argon atmosphere, NaBH_3_CN (0.405 g, 6.45 mmol, 1 equiv.) and TFA (476 µL, 6.45 mmol, 1 equiv.) were added and the pH were adjusted to 5 with NaOH 1 M. The reaction mixture was stirred two additional hours at room temperature. NaOH 1 M was then added to pH 7 and EtOH was evaporated. The product was resuspended in H_2_O (20 mL) and dichloromethane (DCM) (20 mL) and pH was re-adjusted to 9 with NaOH 1 M. The aqueous phase was extracted twice with DCM (2 × 20 mL) and the organic phase was dried over anhydrous sodium sulfate. After DCM evaporation, the product was purified by preparative HPLC with ACN/H_2_O with 0.1 % TFA going from 15/85 to 30/70 in 30 minutes. After solvent evaporation, a yellow oil was obtained with a yield of 27 %.

^1^H NMR (EnPI2CP) (300 MHz, CD_3_OD): δ = 7.40 (m, 1 H, H_Ar_), 7.32 (m, 2 H, H_Ar_), 7.18 (m, 1 H, H_Ar_), 7.01 (m, 2 H, H_Ar_), 6.65- 6.75 (bm, 2 H, H_Ar_), 4.80 (bm, 2 H, N-C**H_2_**-Ph), 4.42 (m, 2 H, N-C**H_2_**-Im), 4.22 (m, 2 H, N-C**H_2_**-Im), 3.87 (s, 3 H, N-C**H_3_**), 3.67 (s, 3 H, N-C**H_3_**), 3.48 (m, 2 H, N-CH-CH-N), 1.4-2.4 (bm, 12 H, N-C**H_2_**-C**H_2_**-CH3 et N-C**H_2,cyclo_**), 0.95 (m, 3 H, N-CH_2_-CH_2_-C**H_3_**).

^13^C NMR (EnIP2CP) (75 MHz, CD_3_OD): δ = 163.3 (C_Ph_-OH), 155.7 (C_quat,Im_), 146.1 (C_quat,Im_), 139.5 (C_Ph_-H), 131.9 (C_Ph_-H), 128.8 (C_Im_H), 125.6 (C_Im_H), 123.2 (C_quatPh_), 121.6 (C_Ph_-H), 119.8 (C_Im_H), 115.9 (C_Im_H), 112.0 (C_Ph_-H), 65.5-63,13 (N-**C**H-**C**H-N, 2C), 55.1 (N-**C**H_2_-CH_2_-CH_3_), 51.4 (N-**C**H**_2_**-Ph), 50.6 (N-**C**H_2_-Im), 45.0 (N-**C**H_2_-Im), 35.3 (N-**C**H_3,_ 2C), 31.9-25,3 (**C**H_2,cyclo_, 4 C), 11.92 (N-CH_2_-CH_2_-**C**H**_3_**)

HRMS (ESI) m/z: [M+H]^+^ Calcd for C_26_H_38_N_6_OH 451.3180. Found 451.318; (Error: 0.0 ppm).

Single isotopic mass : 451.3 m/z.

## Synthesis of EnpI2CP labelled with ^13^C

EnpI2CP labelled with ^13^C was synthetized following the same procedure than that used for EnPI2CP except the use of ^13^C labelled salicylaldehyde as reagent. ^13^C-labelled salicylaldehyde was synthetized by the oxidation of commercial ^13^C-labelled phenol by paraformaldehyde using the protocol described by Yang *et al*. [2].

A mixture of (^13^C_6_) phenol (0.543 g, 5.429 mmol), anhydrous magnesium dichloride (1.312 g, 8.119 mmol), anhydrous triethylamine (2.8 mL) and anhydrous acetonitrile (4.9 mL) was stirred under argon at room temperature for 30 min. Paraformaldehyde (1.315 g, 43.9 mmol) was added to the mixture. The resulting mixture was refluxed for 4 h and then cooled to room temperature. A color change from white to orange was observed. To the mixture was added 10% hydrochloric acid (6.5 mL) to neutralize triethylamine and to reach pH 5. The obtained compound was extracted with diethyl ether (3 × 20 mL). The combined organic layers were washed with distilled water (10 mL) and saturated brine (10 mL), dried over anhydrous Na_2_SO_4_ and filtered off. The solvent was removed by rotary evaporation to produce a brown oil with a yield of 70%.

NMR ^1^H (^13^C-salicylaldehyde) (CDCl_3_, 300 MHz): δ = 11.0 (m, 1H, C_Ar_-CH=O), 9.9 (m, 1H, C_Ar_-OH), 6.5-8 (bm, 4H, H_Ar_). NMR ^13^C (^13^C-salicylaldehyde) (CDCl_3_, 75 MHz): 161 (m, 1 H, C_Ar_), 137 (m, 1 H, C_Ar_), 134 (m, 1 H, C_Ar_), 121-117 (m, 3 H, C_Ar_)

The synthesis of ^13^C-EnPI2CP was performed as for its light analogue, with no intermediate purification. The crude intermediates showed similar NMR-spectra to those obtained for the synthesis of the light analogue of EnPI2CP. The crude final product was purified by preparative HPLC with ACN/H_2_O with 0.1 % TFA going from 15/85 to 30/70 in 30 minutes. As solubility problems were encountered for the purification, only the water-soluble fraction was purified. After solvent evaporation and lyophilization, we obtained the pure heavy ligand as a white solid with a yield of 15%.

NMR ^1^H (^13^C-EnPI2CP) (CD_3_OD, 300 MHz): δ = 7.4 (m, 2H, H_Ar_), 7.31(m, 2H, H_Ar_), 6.98 (m, 2H, H_Ar_), 6.73 (m, 1H, H_Ar_), 6.42 (m, 1H, H_Ar_), 4.45 (m, 2 H, N-C**H_2_**-C_imi_), 4.25 (m, 2 H, N-C**H_2_**-C_imi_), 3.87 (s, 3 H, NC**H_3_**), 3.67 (s, 3 H, NC**H_3_**), 3.49 (m, 2 H, N-C**H_2_**-C_phenol_), 3.2 (m, 2 H, N-C**H_2_**-CH**_2_**-CH_3_), 2.33 (m, 2 H, N-CH_2_-C**H_2_**-CH_3_), 2.02 (m,2 H, C_cyclo_-**H**), 1.81 (m, 4 H, C_cyclo_-**H**), 1.50 (m, 2 H, C_cyclo_-**H**), 0.94 (m, 3 H, N-CH_2_-CH_2_-C**H_3_**). NMR ^13^C (^13^C-EnPI2CP) (CD_3_OD, 75 MHz): 156 (m, 1 C_Ar_), 131 (m, 2 C_Ar_), 121 (m, 2 C_Ar_), 116 (m, 1 C_Ar_)

HRMS (ESI) m/z: [M+H]^+^ Calcd for C_20_^13^C_6_H_38_N_6_OH 457.3381. Found 457.3381; (Error: 0.0 ppm).

The ligands were purified by HPLC and lyophilized. They were dissolved in milliQ water (around 10 mg/mL). An aliquot of the resulting solution was diluted in HEPES buffer (50 mM, pH 7.5) and its concentration was determined by UV-visible titration by successive addition of MnCl2 (10 mM), by following the absorbance at 280 nm. The titration was performed three times. The stock solutions of ligand were diluted to a concentration of 10 mM. Aliquots were prepared and frozen (-20 °C) until used.

Stock solutions of Mn^2+^ (Zn^2+^)-complexes at 10 mM were prepared before each experiment by addition of 1 eq. of anhydrous MnCl_2_ (ZnCl_2_, CuSO_4_, NiCl_2_.6H_2_O, or CoCl_2_) in HEPES buffer (50 mM) to a solution of ligand.

## K_1_ calculation of MnL complexes from UV titration experiments

The protocol is given in the material and method section. The calculation of the theoretical absorbance used to fit the experimental data for K_1_ determination is detailed below.

Table summarizing the extent of the reaction:

Mn^2+^ + L = MnL

|  | $\mathbf{C}_{\mathbf{Mn}}$ | $\mathbf{C}_{\mathbf{L}}$ | $\mathbf{C}_{\mathbf{MnL}}$ |
| --- | --- | --- | --- |
| Initial state | n$\times C_{0}$ | $C_{0}$ | 0 |
| Equilibrium state (intermediate or for n<1) | n $\times C_{0}- C_{0}\times e=(n-e)\times C_{0}$ | $C_{0}-C_{0}\times e =(1-e)\times C_{0}$ | $C_{0}\times$ e |
| Equilibrium state (final or for n>>1) | n $\times C_{0}$ $- C_{0}$ | 0 | $C_{0}$ |

$C_{L}=$ Concentration in ligand (L)

$C_{MnL}=$ Concentration in complex MnL

$C_{Mn}=$ Concentration in Mn(II)

$e=$ extent of the reaction of Mn with L to form MnL after the addition of $n\times C_{0}$ Mn

$K_{1, MnL}=$ Association constant of ligand L with Mn

C_0_ = initial ligand concentration

n = number of molar equivalent of Mn (compared to initial L) added in the solution

$K_{1,MnL}=\frac{C_{MnL}}{C_{L}\times C_{Mn}}$

$$\frac{1}{K_{1,MnL}}= \frac{(n-e)\times C_{0}(1-e)}{e}$$

$\frac{e}{K_{1}}=\left( n-e \right)\times\left( C_{0}-C_{0}e \right)$= $nC_{0}-e{(C}_{0}+nC_{0})+C_{0}e^{2}$

$$n-e(1+n+\frac{1}{C_{0}K_{1}})+e^{2}=0$$

$\Delta={(1+n+\frac{1}{C_{0}K_{1}})}^{2}-4\times n$

$\Delta>0$for $C_{0}$= 40 µM, $K_{1}\approx{10}^{5}-{10}^{6}$ and n between 0 and 3.

Solution of the quadratic equation physically relevant ($e\leq n$):

$$e=\frac{\left( 1+n+\frac{1}{C_{0}K_{1}} \right)-\sqrt{{(1+n+\frac{1}{C_{0}K_{1}})}^{2}-4n\text{ }}\text{ }}{2}$$

$$A_{th}=\varepsilon_{L}C_{L}l+ \varepsilon_{MnL}C_{MnL}l$$

$A_{th}=$ Theoretical absorbance at a fixed wavelength

$\varepsilon_{MnL}=$molar attenuation coefficient of MnL complex at the same fixed wavelength

$\varepsilon_{L}=$molar attenuation coefficient of L at the same fixed wavelength

$l=$ width of the spectrophotometer cuvette.

$$A_{0}=\varepsilon_{L}C_{0}l$$

$$A_{f}=\varepsilon_{MnL}C_{0}l$$

$A_{0}=$ Theoretical initial absorbance of the free ligand solution, no manganese added

$A_{f}=$ Theoretical final absorbance of the MnL solution (all ligands are bound to Mn)

$$A_{th}=A_{0}\times\left( 1-e \right)+A_{f}\times e=A_{0}-e(A_{0}-A_{f})$$

$$A_{th}=A_{0}\times(1-\frac{\left( 1+n+\frac{1}{C_{0}K_{1}} \right)-\sqrt{{(1+n+\frac{1}{C_{0}K_{1}})}^{2}-4n\text{ }}\text{ }}{2})+A_{f}\times\frac{\left( 1+n+\frac{1}{C_{0}K_{1}} \right)-\sqrt{{(1+n+\frac{1}{C_{0}K_{1}})}^{2}-4n\text{ }}\text{ }}{2}$$

## K_1_ calculation of ZnL/Co1CP complexes: competition with the formation of MnL

The association constants of the studied ligands with Zn are very high, so classical UV-vis titration experiments are not sufficient for a precise determination of these values. For this reason, we conducted competitions experiments between the formation of ZnL and MnL for which K_1_ is known. The protocol is detailed in the material and method section of the main article.

Table summarizing the extent of the reaction.

|  | $\mathbf{C}_{\mathbf{Mn}}$ | $\mathbf{C}_{\mathbf{L}}$ | $\mathbf{C}_{\mathbf{MnL}}$ | $\mathbf{C}_{\mathbf{Zn}}$ | $\mathbf{C}_{\mathbf{ZnL}}$ |
| --- | --- | --- | --- | --- | --- |
| Initial state | C_2_ | C_1_ | 0 | $C_{1} \times n_{eq}$ | 0 |
| Equilibrium state (intermediate or for n<1) | ${C_{2}-C}_{1} \times e$ | $C_{1}\times(1-e-e^{'})$ | $C_{1} \times e$ | $C_{1} \times(n_{eq}-e’)$ | $C_{1} \times e'$ |
| Equilibrium state (final or for n>>1) | C_2_ | 0 | 0 | $(n_{eq}-1)\times C_{1}$ | C_1_ |

$$\frac{K_{1, ZnL}}{K_{1,MnL}}=\frac{e'\times C_{1}\times(C_{2}-C_{1}\times e)}{e\times C_{1}\times C_{1}\times(n_{eq}-e^{'})}=\frac{e'\times(C_{2}-C_{1}\times e)}{e\times C_{1}\times(n_{eq}-e^{'})}$$

$C_{X}=$ Concentration in X

$e=$ extent of the reaction of Mn with L to form MnL

$e'=$ extent of the reaction of Zn with L to form ZnL

$K_{1, ZnL}=$ Association constant of ligand L with Zn

$K_{1, MnL}=$ Association constant of ligand L with Mn

C_1_ = initial ligand concentration

C_2_ = initial Mn concentration = $200\times C_{1}$

N_eq_ = number of molar equivalent of Zn (compared to initial L) added in the solution

Assumption: no free ligand in solution (high $K_{1, ZnL}$ and C_1_$\ll$C_2_):

$$e=1-e’$$

$$\frac{K_{1, ZnL}}{K_{1,MnL}}=\frac{e'\times C_{1}\times(C_{2}-C_{1}\times e)}{e\times C_{1}\times C_{1}\times(n_{eq}-e^{'})}=\frac{e'\times C_{1}\times(C_{2}-C_{1}\times(1-e^{'}))}{(1-e^{'})\times C_{1}\times C_{1}\times(n_{eq}-e^{'})}=\frac{e'\times(C_{2}-C_{1}\times(1-e'))}{(1-e')\times C_{1}\times(n_{eq}-e^{'})}=\frac{e^{'}\times C_{2}-{e'\times C}_{1}+C_{1}\times{e^{'}}^{2}}{-\left( e^{'}\times C_{1}\times n_{eq} \right)+\left( {e^{'}}^{2}\times C_{1} \right)+\left( C_{1}\times n_{eq} \right)-\left( {C_{1}\times e}^{'} \right)}$$

$$\left( -\left( e^{'}\times n_{eq} \right)+{e^{'}}^{2}+n_{eq}-e^{'} \right)\times\frac{K_{1, ZnL}}{K_{1,MnL}}=e^{'}\times\frac{C_{2}}{C_{1}}-e'+{e^{'}}^{2}$$

$${e^{'}}^{2}\times\left( 1-\frac{K_{1, ZnL}}{K_{1,MnL}} \right)+e'\times(\frac{C_{2}}{C_{1}}-1+\frac{K_{1, ZnL}}{K_{1,MnL}}\left( n_{eq}+1 \right))-n_{eq}\times\frac{K_{1, ZnL}}{K_{1,MnL}}=0$$

Solution of the quadratic equation:

$$e^{'}=\frac{-\frac{C_{2}}{C_{1}}+1-\frac{K_{1, ZnL}}{K_{1,MnL}}\left( n_{eq}+1 \right)+\sqrt{{(\frac{C_{2}}{C_{1}}-1+\frac{K_{1, ZnL}}{K_{1,MnL}}\left( n_{eq}+1 \right))}^{2}+4\times\left( 1-\frac{K_{1, ZnL}}{K_{1,MnL}} \right)\times n_{eq}\times\frac{K_{1, ZnL}}{K_{1,MnL}}}}{2\times\left( 1-\frac{K_{1, ZnL}}{K_{1,MnL}} \right)}$$

$$A_{th}={\varepsilon_{MnL}C}_{MnL}l+\varepsilon_{ZnL}C_{ZnL}l$$

$A_{th}=$ Theoretical absorbance at a fixed wavelength

$\varepsilon_{MnL}=$molar attenuation coefficient of MnL complex at the same fixed wavelength

$\varepsilon_{ZnL}=$molar attenuation coefficient of ZnL complex at the same fixed wavelength

$C_{ZnL}=$ Concentration in ZnL

$C_{MnL}=$ Concentration in MnL

$l=$ width of the spectrophotometer cuvette.

$$A_{0}=\varepsilon_{MnL}C_{1}l$$

$$A_{f}=\varepsilon_{ZnL}C_{1}l$$

$A_{0}=$ Theoretical initial absorbance of the MnL solution

$A_{f}=$ Theoretical final absorbance of the ZnL solution

$$A_{th}=C_{MnL}\times\frac{A_{0}}{C_{1}}+C_{ZnL}\times\frac{A_{f}}{C_{1}}$$

$$A_{th}=\left( 1-e^{'} \right)\times A_{0}+e^{'}\times A_{f}=A_{0}-e^{'}\times(A_{0}-A_{f})$$

With e’ detailed above

# Supplementary tables

(a)

(b)

Table S1: (a) Pseudo-first order rate constants *k*_obs_ (s^-1^) and (b) half-lives (s) characterizing the metal exchanges occurring between the Mn^2+^ center of the SOD mimics with Zn^2+^, Cu^2+^, Ni^2+^ and Co^2+^. Kinetics of the exchanges were monitored in TRIS (50 mM pH 7.5) using a stopped-flow technique in the presence of 20-fold excess of competitive metal in order to ensure pseudo-first order conditions. The exchanges were observed spectrophotometrically at room temperature and at specific wavelengths chosen to have a high difference in absorbance between the manganese complex and the competitive metal complex. The pseudo-first order rate were then obtained by fitting the theoretical time-absorbance curve to the experimental one using the Biokine32 software. Data represent mean ± SEM for three independent experiments and each independent experiments were performed in duplicates.

|  | [Mn1CP] in lysates  (µM) | Cell concentration in lysates (cells/mL) | n_Mn1CP_ in cells (mol) |
| --- | --- | --- | --- |
| Subculture 1 | 1.38 | 7115000 | 1.9.10^-16^ |
| Subculture 2 | 1.66 | 5537500 | 3.1.10^-16^ |
| Subculture 3 | 2.03 | 5866500 | 3.5.10^-16^ |

Table S2: Quantification of Mn1CP in the lysates of HT29-MD2 cells, stimulated with LPS and incubated with the complex at 100 µM for 6 hours. The quantification was achieved by mass spectrometry by means of a heavy analog of Mn1CP used as a standard. A calibration curve was previously established using this standard (see Figure S28). The molar amount of Mn1CP in each cell was then retrieved by taking into account the dilution executed during the cells’ lysis. The quantification was repeated for 3 independent subcultures with different cells passage numbers. For subculture 1 and 2, the quantification was repeated respectively for 3 and 2 analytical replicates.

# Supplementary figures

(A) (B) (C)


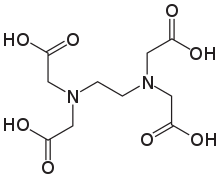


(D) (E) (F) (G)

Figure S1: (A) Structure of EDTA (ethylenediamine tetraacetic acid), (B) Structure of CDTA (cyclohexanediamine tetraacetic acid), (C) Structure of PyC3A, (D) Structure of EnPI2, (E) Structure of EnPI2P, (F) Structure of EnPI2C and (G) Structure of EnPI2CP.

Figure S2: Determination of the association constants K_1_ of the studied SOD mimics from UV-titration experiments. The absorbance of the manganese complexes (288 nm corresponding to the MLCT O_phenolate_ →Mn) have been monitored while adding successively 0.1 equivalent of manganese (concentration in the cuvette equals to 4.5 μM for 0.1 eq) to a solution of ligand at 45 μM in HEPES (50 mM pH 7.6). The initial concentration in ligand was chosen in order to observe an equilibrium in solution at the 1:1 Mn:L ratio and obtain not only the stoichiometry of the reaction Mn(II) + LH —> L(Mn(II)) + H^+^ but also an accurate determination of the association constant. In this purpose, C_ligand_$\times$K_1_ has to be smaller than 100 (see Figure 5 (b) from P. Thordarson *et. al* [3] showing simulated binding isotherms for different C_ligand_$\times$K_1_ ratio’s from 1–10000) [3], which is the case here. Note that when C_ligand_ = 100 K_d_, the complexation equilibrium is shifted towards the complex at 90 % [3]. The absorbance was plotted as a function of the number of added manganese(II) equivalents. The association constants of the manganese complexes were then obtained by fitting the theoretical absorbance curve to the experimental one using the MATLAB curve fitting tool and based on a non-linear regression method. Blue dots correspond to the experimental data and the continuous blue line corresponds to the fit. The calculated K_1_ and the associated Sum of Squares Errors (SSE) are mentioned on each plot.


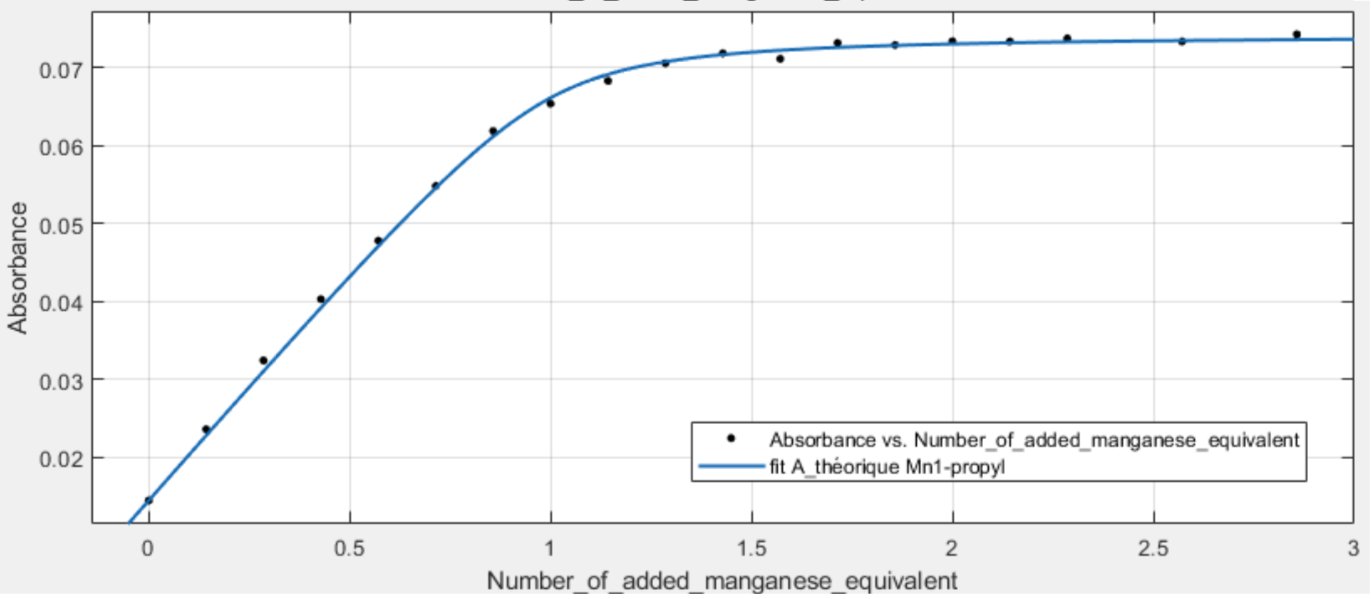

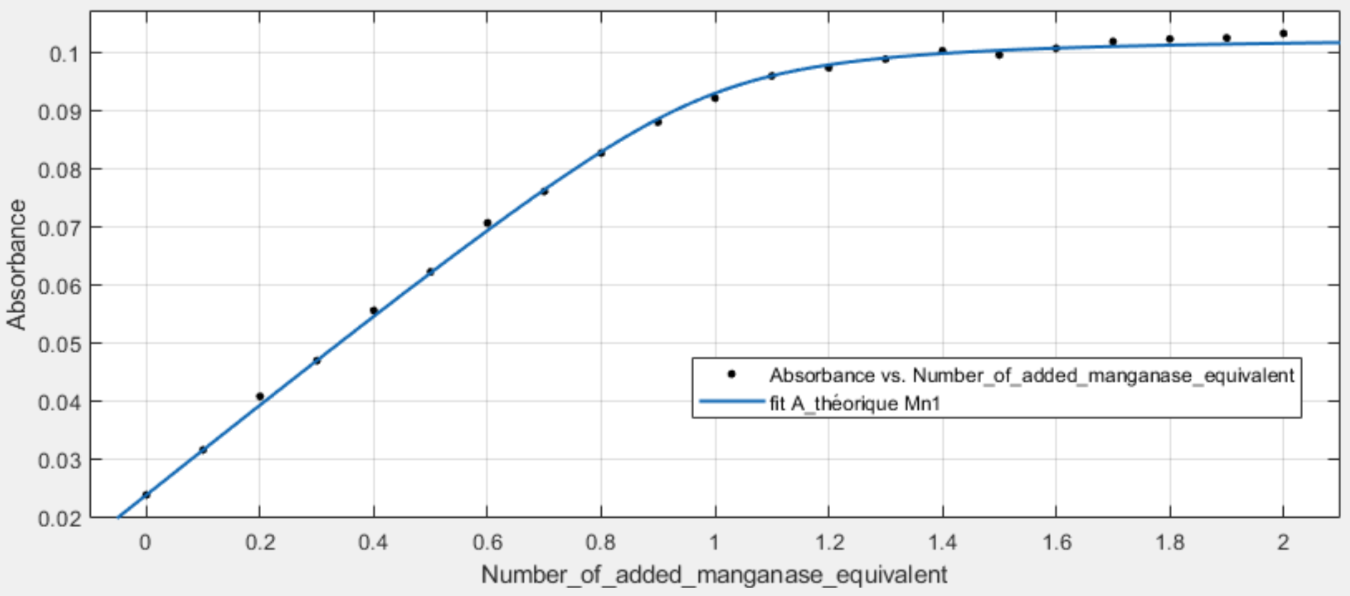

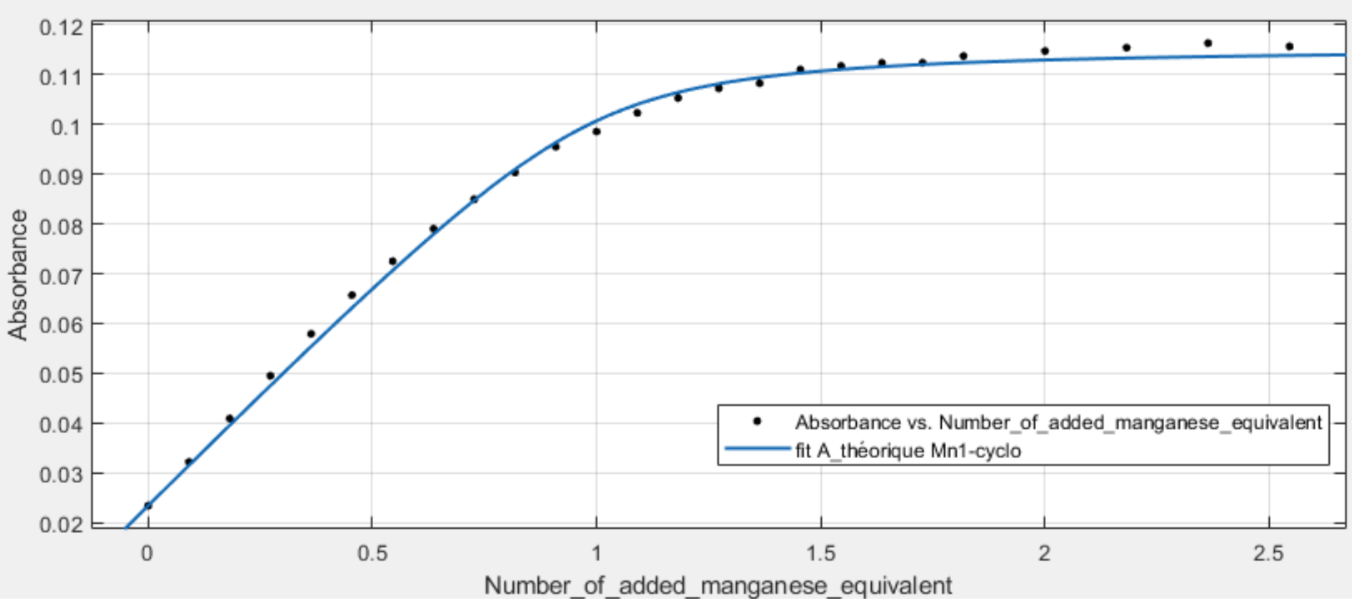

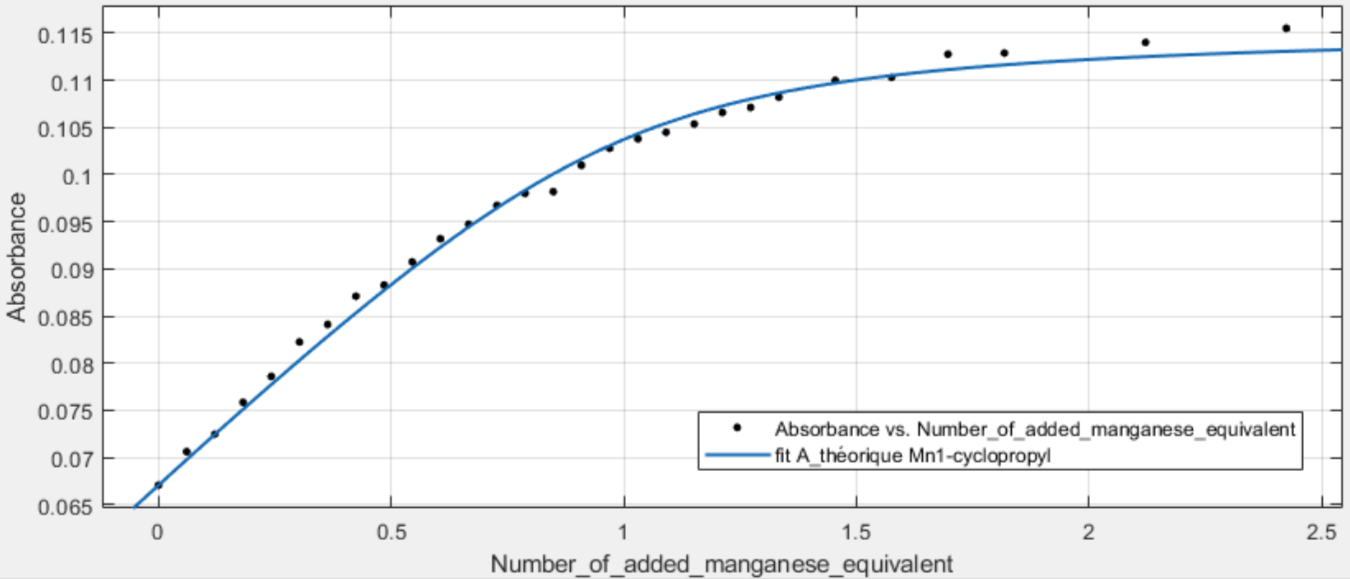

$$\mathbf{K}_{\boldsymbol{1\_Mn1}}\mathbf{=1.22}\boldsymbol{\pm0.18\times}\mathbf{10}^{\mathbf{6}}\mathbf{M}^{\mathbf{-1}}$$

$$\mathbf{K}_{\mathbf{1}_{\mathbf{Mn1}}\boldsymbol{P}}\mathbf{=1.035}\boldsymbol{\pm1.795\times}\mathbf{10}^{\mathbf{5}}\mathbf{M}^{\mathbf{-1}}$$

$$\mathbf{K}_{\mathbf{1}_{\mathbf{Mn1}}\boldsymbol{C}}\mathbf{=0.7093}\boldsymbol{\pm1.141\times}\mathbf{10}^{\mathbf{5}}\mathbf{M}^{\mathbf{-1}}$$

$$\mathbf{K}_{\mathbf{1}_{\mathbf{Mn1}}\mathbf{CP}}\mathbf{=0.2838}\boldsymbol{\pm0.329\times}\mathbf{10}^{\mathbf{5}}\mathbf{M}^{\mathbf{-1}}$$


Figure S3: UV-visible spectra of (A) Mn1 and the Zn(II) complex Zn-EnPI2 labelled Zn1, (B) Mn1P and the Zn(II) complex Zn-EnPI2P labelled Zn1P, (C) Mn1C and the Zn(II) complex Zn-EnPI2C labelled Zn1C and (D) Mn1CP and the Zn(II) complex Zn-EnPI2CP labelled Zn1CP. The solutions of complexes were prepared at 100 µM in HEPES 50 mM pH 7.6, by addition of 1:1 ligand: + MnCl_2_ or ZnCl_2_.

Figure S4: UV-visible spectra of (A) Mn1 and the Cu(II) complex Cu-EnPI2 labelled Cu1, (B) Mn1P and the Cu(II) complex Cu-EnPI2P labelled Cu1P, (C) Mn1C and the Cu(II) complex Cu-EnPI2C labelled Cu1C and (D) Mn1CP and the Cu(II) complex Cu-EnPI2CP labelled Cu1CP. The solutions of complexes were prepared at 100 µM in HEPES 50 mM pH 7.6 by addition of 1:1 ligand: + MnCl_2_ or CuSO_4_.

Figure S5: UV-visible spectra of (A) Mn1 and the Ni(II) complex Ni-EnPI2 labelled Ni1, (B) Mn1P and the Ni(II) complex Ni-EnPI2P labelled Ni1P, (C) Mn1C and the Ni(II) complex Ni-EnPI2C labelled Ni1C and (D) Mn1CP and the Ni(II) complex Ni-EnPI2CP labelled Ni1CP. The solutions of complexes were prepared at 100 µM in HEPES 50 mM pH 7.6 by addition of 1:1 ligand: + MnCl_2_ or NiCl_2_.

Figure S6: UV-visible spectra of (A) Mn1 and the Co(II) complex Co-EnPI2 labelled Co1, (B) Mn1P and the Co(II) complex Co-EnPI2P labelled Co1P, (C) Mn1C and the Co(II) complex Co-EnPI2C labelled Co1C and (D) Mn1CP and the Co(II) complex Co-EnPI2CP labelled Co1CP. The solutions of complexes were prepared at 100 µM in HEPES 50 mM pH 7.6 by addition of 1:1 ligand: + MnCl_2_ or CoCl_2_.

Figure S7: UV-vis kinetics study of the metal exchanges occurring between the manganese center of the SOD mimics and respectively (A) Zn^2+^ ions, (B) Cu^2+^ ions, (C) Ni^2+^ ions and (D) Co^2+^ ions present at one equivalent in solution (addition of ZnCl_2_, CuSO_4_, NiCl_2_.6H_2_O, or CoCl_2_). The percentage of complexes that underwent metal exchanges was monitored spectrophotometrically by following the absorbance at a wavelength, chosen to have a noticeable difference in absorbance between the manganese complex and the exchanging ion complex: 300 nm for Zn^2+^ exchanges, 265 nm for Cu^2+^ exchanges, 300 nm for Co^2+^ exchanges (except 280 nm for Co1CP), 265 nm for Ni^2+^ exchanges. The spectra of the Mn(II) complexes and the exchanging ions complexes are given in Figure S3, S4, S5 et S6 and were used to choose these monitoring wavelengths. A solution of the SOD mimic was prepared at 0.1 mM in TRIS buffer (50 mM buffer pH 7.5) in a semi-microcuvette (1.5 mL). One equivalent of competitive metal was then added in the microcuvette and after a quick stirring, the absorbance was measured for around 10 minutes. For (A), (B) and (D), the experiments were performed at 5°C as the metal exchanges were too fast at 25°C preventing any kinetic study. For Ni^2+^ exchanges study, the temperature was maintained at 25°C.

Figure S8: HPLC analysis of the four EnPI2-derivated ligands on a C_18_ column using a gradient of ACN in H_2_O from 5 % to 50 % in water over 10 minutes. Both solvents contained TFA (0.1 %). Pool: the four ligands were pooled at 1 mM and injected altogether in the chromatographic system. They were also injected separately at 1 mM to assign the peaks previously visualized in the pooled sample.

Figure S9: Cyclic voltammograms of the SOD mimics at 100 μM in HEPES (50 mM, pH 7.5, ionic strength = 12.5 mM) at a glassy carbon working electrode (3 mm diameter) with a scan rate of 500 mV/sec. Experiments are carried out under an argon stream at room temperature. The electrochemical apparatus contained a platinum wire counter electrode and an aqueous calomel electrode saturated with KCl (SCE) as the reference electrode (0.241 V + SCE = normal hydrogen electrode).


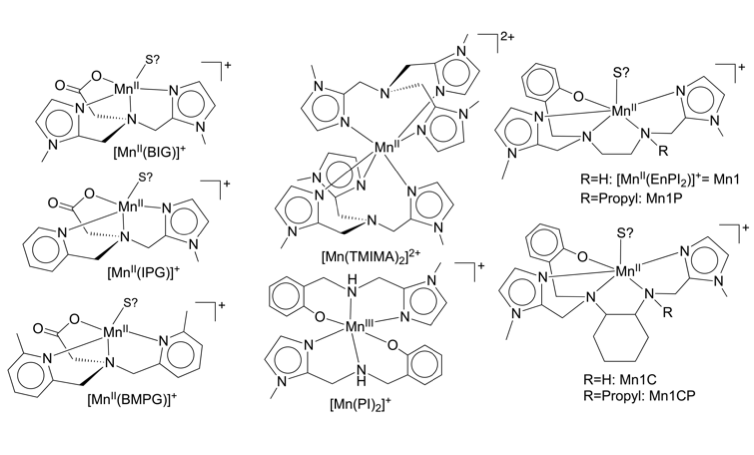


Figure S10: Mn(II)-complexes bio-inspired from SOD. See also [4].

Figure S11: McCord and Fridovich assays of SOD mimics performed at pH 7.4 in HEPES 50 mM with the use of XTT as redox competitive indicator. [XTT ]= 100 µM. The XTT absorbance at 470 nm is measured over time in absence and in presence of SOD mimics. The slope of the absorbance increase is measured in absence (p1) and in presence (p2) of the SOD mimic.

(A) The ratio (p1-p2)/p2 is plotted as a function of the SOD mimics concentration (dots). The IC_50_ can be extrapolated from the linear regression of this curve (line) by determining the concentration for which (p1-p2)/p2 is equal to 1 (𝑝1 = 2 ∗ 𝑝2).

(B) Table classifying the SOD mimics IC_50_ and their catalytic constants for superoxide dismutation. IC_50_ is the concentration in SOD mimic at which the reaction rate of the XTT indicator is reduced of 50 % that without any SOD mimic. k_cat_ values were calculated from that of XTT (equal to 2.9.10^4^ M^-1^s^-1^) and from the SOD mimics IC_50_: k_cat_ = k_XTT_*[XTT]/IC_50_ [4], [5]. The lower the IC_50_ for the SOD mimics, the higher is the catalytic rate constant for superoxide dismutation. The experiments were repeated twice and the results given here represent the mean ± the standard deviation.

### Figure S12: Evaluation of the antioxidant and anti-inflammatory activity of Mn1, Mn1C, Mn1P and Mn1CP in intestinal epithelial cells HT29-MD2 activated with LPS (0.1 μg/mL)

(a,d,g,j) Bottom: representative Western blots of MnSOD expression in HT29−MD2 cells incubated for 6 hours with (a) Mn1, (b) Mn1C, (c) Mn1P and (d) Mn1CP at different concentrations, without (-) and with (+) LPS. Top: stain-free blot imaging showing the total proteins for the same samples.

(b,e,h,k) Quantification of MnSOD expression. MnSOD expression was measured by Western blot in lysates of LPS-activated HT29−MD2 cells incubated for 6 hours with (b) Mn1, (e) Mn1P, (h) Mn1C and (k). Mn1CP at different concentrations. The MnSOD expression intensity measured for activated cells was set at 100 %. The abundances of MnSOD were normalized to the total amount of protein in each lane (top panels a,d,g,j).

(c,f,i,l) Quantification of the inflammatory marker IL-8. IL-8 secretion was measured by ELISA in supernatant of HT29−MD2 cells incubated for 6 hours with (c) Mn1, (f) Mn1P, (i) Mn1C and (l) Mn1CP at different concentrations, without (-) and with (+) LPS and compared to MnCl_2_ (100 µM)

MnSOD expression and IL-8 secretion data represent mean ± SEM for at least three independent experiments: the number of independent experiments is indicated above each column. The p-values were calculated using the student test (bilateral test with equal variances not assumed). The mean rank of each column was compared to that of the LPS control, each comparison stands alone. (***) p < 0.001, (**) p < 0.01 and (*) p < 0.05 versus LPS control, and ns means non-significant. Without LPS, no significant differences were observed between all of these conditions (see Figure S15).

Figure S13: Evaluation of the antioxidant activity of MnCl_2_ incubated at 100 µM in intestinal epithelial cells HT29-MD2 activated with LPS (0.1 μg/mL). Bottom: representative Western blot of MnSOD expression in HT29−MD2 cells incubated for 6 hours with MnCl_2_ at 100 µM with (+) LPS. Top: stain-free blot imaging showing the total proteins for the same samples. Red cross: lane of the gel that is not commented here.

By setting the MnSOD expression intensity measured for LPS control at 100 %, the intensity for MnCl_2_100 µM is equal to 98% that of the LPS control. MnCl_2_do not have any effect on the LPS-induced overexpression of MnSOD when incubated at 100 µM. This confirms the requirement of the manganese (II) complexation. The ligands allow to tune the redox potential of Mn(III)/Mn(II) in the appropriate range to obtain SOD activity. The abundance of MnSOD were normalized to the total amount of protein in each lane.

Figure S14: Evaluation of the cytotoxicity induced by the SOD mimics using the LDH assay. LPS-activated HT29-MD2 cells were incubated with the SOD mimics at 100 µM for 6 hours and the cytotoxicity of the compounds was tested by following the release of the cytosolic lactate dehydrogenase (LDH) into the supernatant, indicative of cell membrane damages. LDH activity can be measured based on LDH ability to catalyze the reduction of pyruvate into lactate in presence of NADH absorbing at 340 nm, which is oxidized to form NAD^+^. The decrease in absorbance at 340nm due to NADH consumption can be assimilated to LDH activity and eventually the percentage of LDH release can be obtained by dividing the LDH activity in supernatant by the sum of activity in supernatants and cell lysates. Data represent the % of LDH released extracellularly (mean of at least 2 independent experiments). The percentage of LDH released in supernatants were always below 10% (which were chosen as the limit of noncytotoxicity) and in average below 5% for all SOD mimics. We can conclude that none of the SOD mimics induce meaningful cytotoxicity at 100 µM in HT29-MD2 cells when prepared with a 1.4:1 manganese-to-ligand ratio 2 hours before adding to the culture medium.

Figure S15: Quantification of IL-8 secretion and of MnSOD expression in controls without LPS. MnSOD expression was measured by Western blot in lysates of non-activated HT29−MD2 cells incubated with the four SOD mimics for 6 hours. The MnSOD expression intensity measured for activated cells is set at 100 %. The abundance of MnSOD were normalized to the total amount of protein in each lane. IL-8 secretion was measured by ELISA in supernatant of non-activated HT29−MD2 cells incubated for 6 hours with the four SOD mimics at 100 µM. The IL-8 amount measured for activated cells is set at 100 %.

(a) and (b) Data represent mean ± SEM for at least two independent experiments (except only one independent experiment for (a)-Mn1P). (c) Representative western blot analysis of MnSOD expression in controls without LPS. Red crosses: lane of the gel that are not commented here

### Figure S16: Evaluation of the anti-inflammatory activity and of the cytotoxicity of the four zinc complexes in intestinal epithelial cells activated with LPS (0.1 μg/mL)

1. Quantification of the inflammatory marker IL-8 by ELISA in supernatant of LPS-activated HT29−MD2 cells incubated for 6 hours with the Zn(II) complexes at 10 µM. The IL-8 amount measured for activated cells is set at 100 %. Data represent mean ± SEM: the number of independent experiments is indicated above each column. Each independent experiments was performed in duplicates. The p-values were calculated using the student test (bilateral test with equal variances not assumed). The mean rank of each column was compared to that of the LPS control, each comparison stands alone. (***) p < 0.001, (**) p < 0.002 and (*) p < 0.033 versus LPS control, and ns means non-significant.
2. The cytotoxicity of the Zn(II) complexes at 10 µM in LPS-activated HT29-MD2 cells was tested by using the LDH assay. Data represent the % of LDH released extracellularly (mean of 2 independent experiments). The percentage of LDH released in supernatants were always below 10 % and in average below 5% for all zinc complexes mimics, showing that they do not induce meaningful cytotoxicity.

Figure S17: McCord and Fridovich assays of Cu-complexes and free Cu^2+^ performed at pH 7.4 in HEPES 50 mM with the use of XTT as redox competitive indicator. [XTT]= 100 µM. The XTT absorbance at 470 nm is measured over time in absence and in presence of Cu-complexes. The slope of the absorbance increase is measured in absence (p1) and in presence (p2) of the Cu-complexes. The ratio (p1-p2)/p2 is plotted as a function of the Cu-complex concentration (dots). The IC_50_ can be extrapolated from the linear regression of this curve (line) by determining the concentration for which (p1-p2)/p2 is equal to 1 (𝑝1 = 2 ∗ 𝑝2). Calculated IC_50_ are given in red on each graph.

Figure S18: Statistical results of manganese quantification by ICP-MS in LPS-activated HT29-MD2 cells incubated with the 4 studied SOD mimics at 10µM and 100µM for 6 hours. A two-by-two comparison was achieved by using a student test (bilateral test with equal variances not assumed). The calculated p-values are given in the right table. Asterisk annotations for significance are given in the left table with the following threshold level (***) p < 0.001, (**) p < 0.01 and (*) p < 0.05.

Figure S19: Determination of the association constants K_1_ of the studied ligands with Zn(II) from competition experiments in HEPES 50 mM pH 7.5. The direct measure of K_1_ for Zn(II) complexes was not possible because the K_1_ values are too high which would impose to use a very low concentration for the titration at which the absorbance is too small to be precisely measured by a classical spectrophotometer. The absorbance of the Zn(II) complex have been monitored while adding successively 0.1 equivalent of Zn(II) (ZnCl_2_) to a solution of manganese complex at 10 μM.

The initial concentration in manganese complex is very low in order to observe an equilibrium in solution and obtain not only the stoichiometry but also the association constant. The initial solution was prepared by adding 200 equivalents of manganese (2 mM) to a solution of ligand at 10 µM. The addition of a large excess of manganese is necessary to 1) make the assumption that the concentration of ligand is null (see calculation of K_1,ZnL_ in supplementary information), 2) to shift the equilibrium to the formation of the Mn(II) complex and disfavored the Zn(II) complex that is very thermodynamically favored.

The absorbance is plotted as a function of the number of added Zn(II) equivalents. The association constant of the zinc complexes were then obtained by fitting the theoretical absorbance curve to the experimental one using the MATLAB curve fitting tool based on a non-linear least-square regression method (see calculation of K_1,ZnL_ in supplementary information). Dots correspond to the experimental data and the continuous line correspond to the fit. The calculated K_1_ and the associated Sum of Squares Errors (SSE) are mentioned on each plot.

Figure S20: Mass spectrum of Mn1 diluted at 0.2 µM in 20% NH_4_CO_3_/ 80% ACN. The figure was zoomed onto the m/z region containing the studied complex.


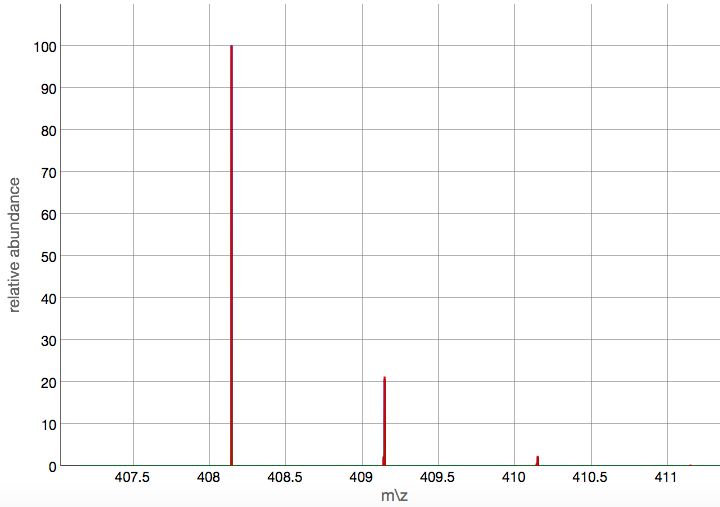

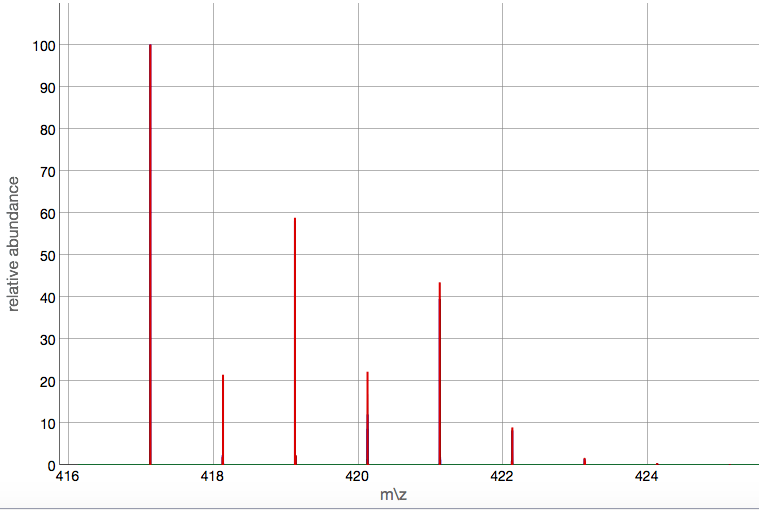


### Figure S21: Predicted isotopic patterns of Mn1 (left) and Zn1 (right).

Figure S22: Mass spectrum of Mn1P diluted at 2 µM in 20% NH_4_CO_3_/ 80% ACN. The figure was zoomed onto the m/z region containing the studied complex.


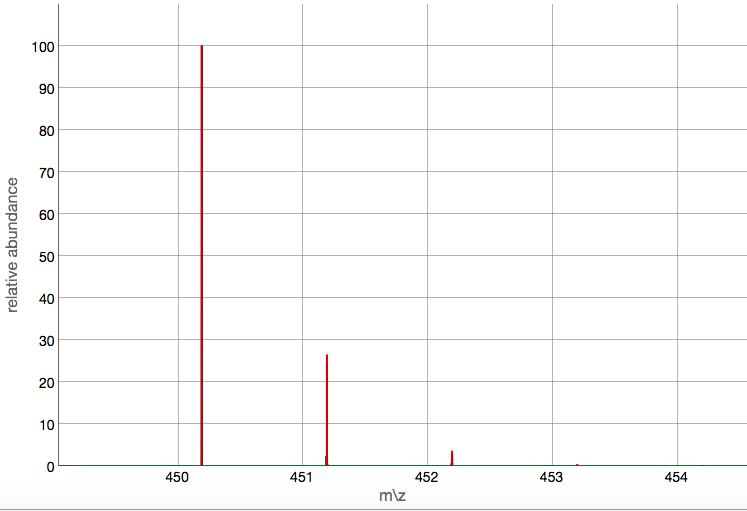

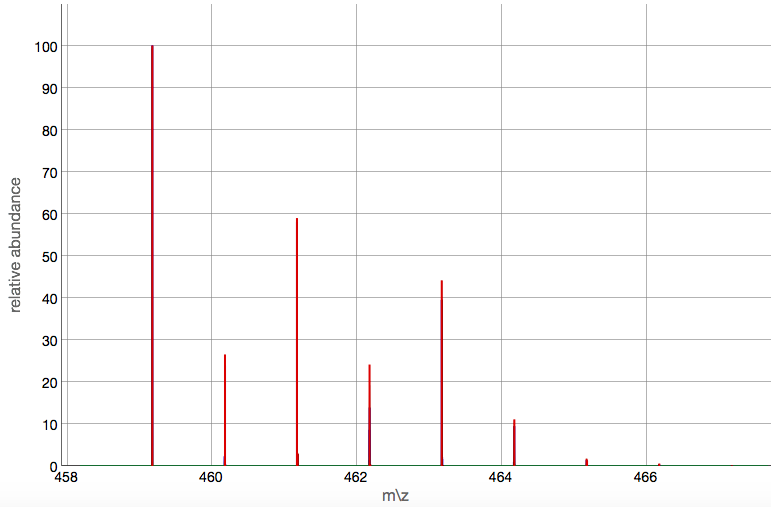


### Figure S23: Predicted isotopic patterns of Mn1P (left) and Zn1P (right).

Figure S24: Mass spectrum of Mn1C diluted at 0.2 µM in 20% NH_4_CO_3_/ 80% ACN. The figure was zoomed onto the m/z region containing the studied complex.


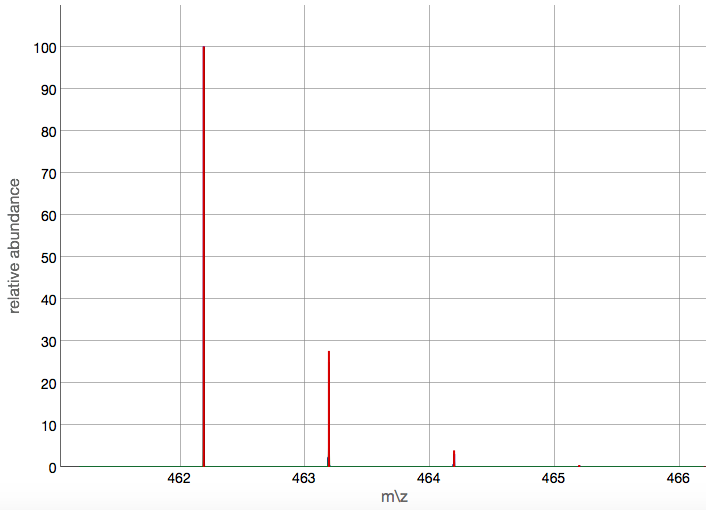

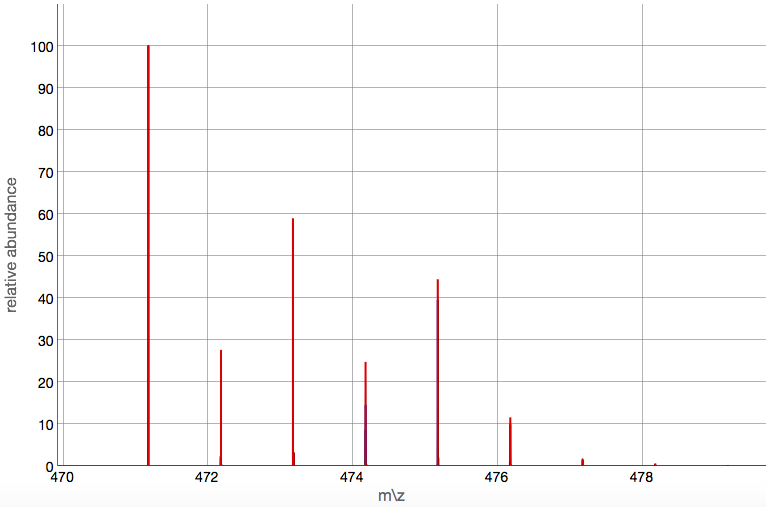


### Figure S25: Predicted isotopic patterns of Mn1C (left) and Zn1C (right).

Figure S26: Mass spectrum of Mn1CP diluted at 0.2 µM in 20% NH_4_CO_3_/ 80% ACN. The figure was zoomed onto the m/z region containing the studied complex.


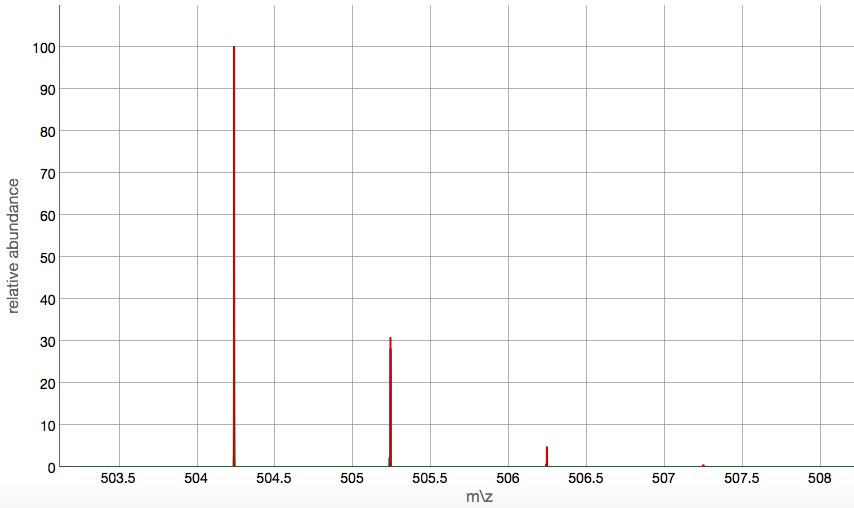

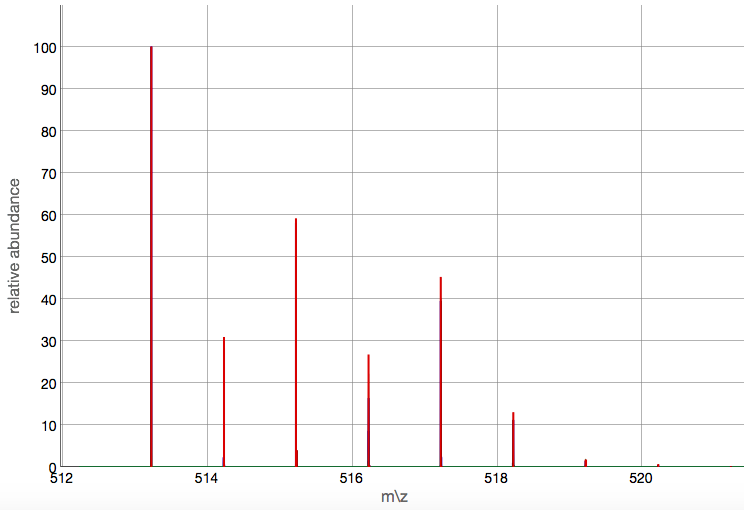


### Figure S27: Predicted isotopic patterns of Mn1CP (left) and Zn1CP (right).

Figure S28: Determination of the association constants K_1_ of EnpI2CP ligand with Co(II) from competition experiments with Mn1CP in HEPES (50 mM pH 7.5). The direct measure of K_1_ for Co(II) complexes is not possible because the K_1_ value is too high which would impose to use a very low concentration for the titration, at which the absorbance would be too small to be precisely measured by a classical spectrophotometer. The absorbance of the Co(II) complex have been monitored while adding successively 0.1 equivalent of Co(II) (CoCl_2_) to a solution of manganese complex at 40 μM.

The initial concentration in manganese complex is very low in order to observe an equilibrium in solution and obtain not only the stoichiometry but also the association constant. The initial solution was prepared by adding 50 equivalents of manganese (2 mM) to a solution of ligand at 40 µM. The addition of a large excess of manganese is necessary to 1) make the assumption that the concentration of ligand is null, 2) to hinder the formation of the cobalt complexes that is very thermodynamically favored.

The absorbance is plotted as a function of the number of added Co(II) equivalents. The association constant of the Co(II) complexes were then obtained by fitting the theoretical absorbance curve to the experimental one using the MATLAB curve fitting tool based on a non-linear least-square regression method. Dots correspond to the experimental data and the continuous line correspond to the fit. The calculated K_1_ and the associated Sum of Squares Errors (SSE) are mentioned on the plot.

Figure S29: Calibration curves for Mn1CP quantification obtained in cell lysates diluted in 20% NH_4_CO_3_/ 80% ACN. The calibration curve was obtained by spiking LPS-stimulated HT29-MD2 lysates with the standard at 0.4 µM and with Mn1CP at concentrations varying between 0.05 µM to 0.8 µM. The ratio of the signal intensity of Mn1CP on that of the standard were plotted as a function of Mn1CP concentration. The signal intensity of both Mn1CP and the standard were obtained by summing the peak intensity of the complexes and their first isotopes.


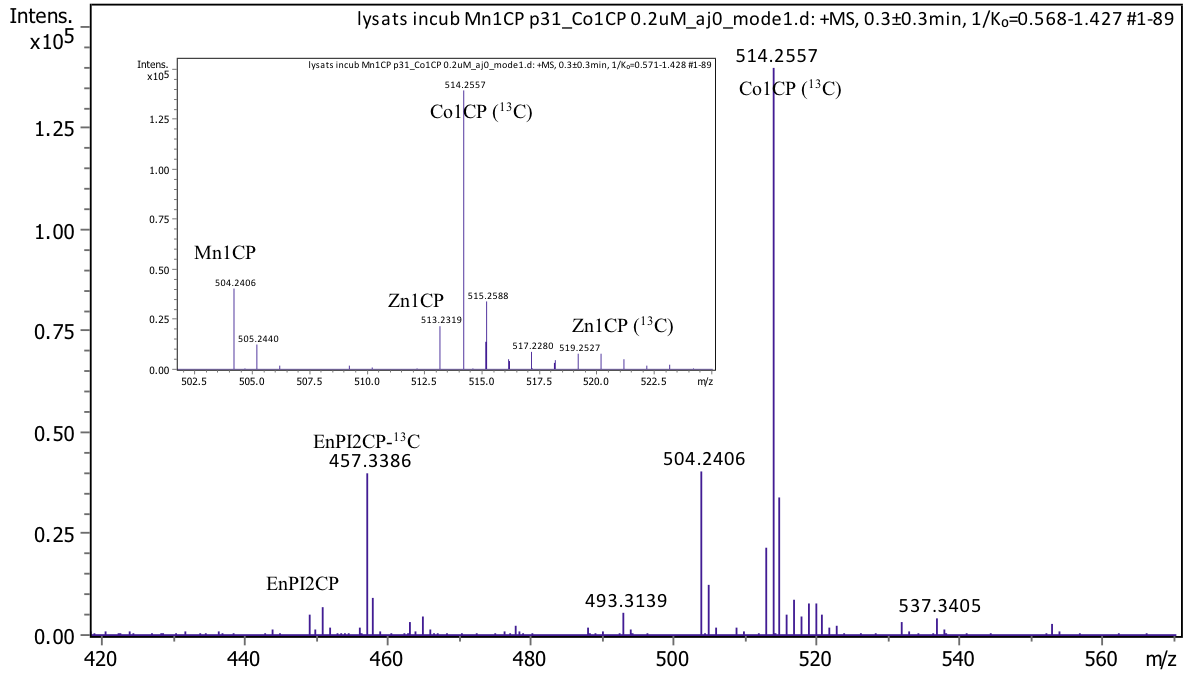


Figure S30: Examples of mass spectra of a LPS-stimulated HT29-MD2 lysates, previously incubated with the SOD mimic Mn1CP for 6 hours and ultra-centrifuged at 100,000 rpm for 20 minutes. The lysates (10-15 10^6^ cells in 2 mL in NH_4_CO_3_ (50 mM) were diluted in 20% NH_4_CO_3_/ 80% ACN and spiked with the standard Co1CP (^13^C) at 0.4 µM. The figures were zoomed onto the m/z region containing the studied complexes. The peak corresponding to Mn1CP, Co1CP (^13^C) and their isotopic pattern are clearly visible and annotated on the spectrum. Zn1CP (^13^C) displays peaks with relatively low intensity and was neglected for the quantification.

Figure S31: Quantification of Mn1CP in LPS-stimulated HT29-MD2 lysates by the method of standard additions. The lysates were beforehand ultra-centrifuged at 100,000 rpm for 20 minutes, diluted in 20% NH_4_CO_3_/ 80% ACN and spiked with the standard Co1CP (^13^C) at 0.4 µM. Mn1CP was spiked at known concentration (0.2 µM and 0.4 µM) in the lysates of cells previously incubated with Mn1CP at 100 µM for 6 hours. The equation of the linear regression and the R-squared value of the regression are indicated on the graph. By taking into account the dilution done for the analysis and the number of cells in the lysate, an intracellular molar amount of 2.69.10^-16^ mol of Mn1CP per cells was measured using this method. This is close to the values obtained with the previous quantification. This result validates our approach based on the use of a heavy analog of Mn1CP as a standard and on the establishment of a calibration curve to quantify the SOD mimic inside cells.

# Abbreviations

BCA Bicinchoninic acid
H/BSA Human/Bovine serum albumin

CDTA cyclohexanediamine tetraacetic acid

EDTA Ethylenediamine tetraacetic acid
ELISA Enzyme-linked immunosorbent assay
EPR Electron paramagnetic resonance
HEPES 4-(2-hydroxyethyl)-1-piperazineethanesulfonic acid
HPLC High performance liquid chromatography
HRP Horse-radish peroxidase
IBDs Inflammatory Bowel Diseases
ICP-MS Inductively coupled plasma mass spectrometry

IL-8 Interleukin 8
LDH Lactate dehydrogenase
LPS Lipopolysaccharides
MnSOD Manganese superoxide dismutase
NADH Nicotinamide adenine dinucleotide
NHE Normal hydrogen electrode
PBS Phosphate buffer saline
ROS Reactive oxygen species
SCE Saturated calomel electrode (saturated with KCl)
SODs Superoxide Dismutases

TFA trifluoroacetic acid
TIMS Trapped Ion Mobility Spectrometry
TLR-4 Toll-like receptors
TOF-MS Time of flight mass spectrometry
WB Western Blot
XTT 2,3-bis-(2-methoxy-4-nitro-5-sulfophenyl)-2H-tetrazolium-5-carboxanilide

[1] F. Cisnetti *et al.*, « A New Pentadentate Ligand Forms Both a Di- and a Mononuclear MnII Complex: Electrochemical, Spectroscopic and Superoxide Dismutase Activity Studies », *Eur. J. Inorg. Chem.*, vol. 2007, n^o^ 28, p. 4472‑4480, oct. 2007, doi: 10.1002/ejic.200601236.

[2] Z.-M. Yang et L. Lu, « Synthesis of deuterated herbicidal ZJ0273, ZJ0702, ZJ0777, and SIOC0163 », *J Label Compd Radiopharm*, p. n/a-n/a, 2010, doi: 10.1002/jlcr.1749.

[3] P. Thordarson, « Determining association constants from titration experiments in supramolecular chemistry », *Chem. Soc. Rev.*, vol. 40, n^o^ 3, p. 1305‑1323, 2011, doi: 10.1039/C0CS00062K.

[4] C. Policar, « Mimicking SOD, Why and How: Bio-Inspired Manganese Complexes as SOD Mimic », in *Redox-Active Therapeutics*, 2016, p. 125‑164.

[5] S. Durot, F. Lambert, J.-P. Renault, et C. Policar, « A Pulse Radiolysis Study of Catalytic Superoxide Radical Dismutation by a Manganese(II) Complex with an N-Tripodal Ligand », *Eur. J. Inorg. Chem.*, vol. 2005, n^o^ 14, p. 2789‑2793, juill. 2005, doi: 10.1002/ejic.200400834.
